# A titin truncating variant linked to atrial fibrillation increases atrial profibrotic signalling and cholinergic sensitivity

**DOI:** 10.1093/cvr/cvag112

**Published:** 2026-05-27

**Authors:** Max J Cumberland, Albert Dasí, Naeramit Sontayananon, Alan D Marcus, Alex R Qin, Leto Riebel, Jonas Euchner, Amar J Azad, Caitlin Hall, Christopher O’Shea, James G W Smith, Charikleia Papadopoulou, Eric A Miska, Davor Pavlovic, Ellis Patrick, James J H Chong, Paulus Kirchhof, Chris Denning, Benjamin Davies, Blanca Rodriguez, Andrew P Holmes, Katja Gehmlich

**Affiliations:** Department of Cardiovascular Science, School of Medical Sciences, University of Birmingham, Edgbaston B15 2TT, UK; Centre for Heart Research, The Westmead Institute for Medical Research, University of Sydney, 176 Hawkesbury Road, Westmead, NSW 2145, Australia; Department of Computer Science, British Heart Foundation Centre of Research Excellence Oxford, University of Oxford, Oxford, UK; Division of Cardiovascular Medicine, Radcliffe Department of Medicine, and British Heart Foundation Centre of Research Excellence Oxford, University of Oxford, Oxford OX3 9DU, UK; Centre for Heart Research, The Westmead Institute for Medical Research, University of Sydney, 176 Hawkesbury Road, Westmead, NSW 2145, Australia; Centre for Cancer Research, The Westmead Institute for Medical Research, The University of Sydney, Sydney, New South Wales, Australia; Department of Computer Science, British Heart Foundation Centre of Research Excellence Oxford, University of Oxford, Oxford, UK; Department of Cardiovascular Science, School of Medical Sciences, University of Birmingham, Edgbaston B15 2TT, UK; Centre of Membrane Proteins and Receptors (COMPARE), The Universities of Birmingham and Nottingham, The Midlands B15 2TT, UK; Department of Cardiovascular Science, School of Medical Sciences, University of Birmingham, Edgbaston B15 2TT, UK; Department of Cardiovascular Science, School of Medical Sciences, University of Birmingham, Edgbaston B15 2TT, UK; Department of Cardiovascular Science, School of Medical Sciences, University of Birmingham, Edgbaston B15 2TT, UK; Heart Rhythm Research Group, University of Warwick, Coventry CV2 2DX, UK; Centre for Metabolic Health, Norwich Medical School, University of East Anglia, Norwich Research Park, Norwich NR4 7UQ, UK; Department of Biochemistry, University of Cambridge, Sanger Building, 80 Tennis Ct Rd, Cambridge CB2 1GA, UK; Royal Papworth Hospital, Papworth Rd, Trumpington, Cambridge CB2 0AY, UK; Department of Biochemistry, University of Cambridge, Sanger Building, 80 Tennis Ct Rd, Cambridge CB2 1GA, UK; Royal Papworth Hospital, Papworth Rd, Trumpington, Cambridge CB2 0AY, UK; Department of Cardiovascular Science, School of Medical Sciences, University of Birmingham, Edgbaston B15 2TT, UK; Centre for Cancer Research, The Westmead Institute for Medical Research, The University of Sydney, Sydney, New South Wales, Australia; Centre for Heart Research, The Westmead Institute for Medical Research, University of Sydney, 176 Hawkesbury Road, Westmead, NSW 2145, Australia; Department of Cardiovascular Science, School of Medical Sciences, University of Birmingham, Edgbaston B15 2TT, UK; Department of Cardiology, University Heart and Vascular Center Hamburg, University Medical Center Hamburg-Eppendorf, Hamburg, Germany; German Center for Cardiovascular Research (DZHK), partner Site Hamburg/Kiel/Lübeck, Hamburg, Germany; University of Nottingham, Biodiscovery Institute, University Park, Nottingham, NG7 2RD, UK; Wellcome Centre for Human Genetics, University of Oxford, Roosevelt Drive, Headington, Oxford OX3 7BN, UK; Department of Computer Science, British Heart Foundation Centre of Research Excellence Oxford, University of Oxford, Oxford, UK; Department of Cardiovascular Science, School of Medical Sciences, University of Birmingham, Edgbaston B15 2TT, UK; Department of Biomedical Sciences, School of Infection, Inflammation and Immunology, University of Birmingham, Edgbaston B15 2TT, UK; Department of Cardiovascular Science, School of Medical Sciences, University of Birmingham, Edgbaston B15 2TT, UK; Division of Cardiovascular Medicine, Radcliffe Department of Medicine, and British Heart Foundation Centre of Research Excellence Oxford, University of Oxford, Oxford OX3 9DU, UK

**Keywords:** Titin, Atrial fibrillation, TTNtv, hiPSC-CMs, EHTs, AF, Atrial fibrosis

## Abstract

**Aims:**

Titin truncating variants (TTNtv) are a major genetic cause of dilated cardiomyopathy (DCM), accounting for approximately 25% of familial cases. Atrial fibrillation (AF) frequently occurs in DCM patients carrying TTNtv and may precede overt ventricular dysfunction, suggesting an atrial-specific disease mechanism. How TTNtv increase susceptibility to AF, particularly in the absence of established DCM, remains incompletely understood. This study aimed to define the cellular and molecular mechanisms by which a clinically relevant TTNtv predisposes to atrial arrhythmogenesis.

**Methods and results:**

We introduced a patient-associated TTNtv (*TTN* c.59926+1G>A) into human induced pluripotent stem cell-derived atrial cardiomyocytes (hiPSC-CMs). TTNtv hiPSC-CMs exhibited proarrhythmic electrophysiological alterations, including increased spontaneous beating frequency, abnormal sodium channel kinetics, and heightened sensitivity to cholinergic agonists. *In silico* simulations demonstrated that heightened cholinergic sensitivity was sufficient to trigger AF under conditions of sinus tachycardia. RNA sequencing revealed dysregulation of sarcomere assembly and extracellular matrix pathways, and TTNtv hiPSC-CMs showed structurally shortened sarcomeres. Engineered heart tissues composed of TTNtv hiPSC-CMs co-cultured with cardiac fibroblasts demonstrated reduced contractile force and increased secretion of collagen, fibronectin-1 and TGF-β1, consistent with activation of profibrotic signalling. Together, these findings indicate that a TTNtv can cause intrinsic atrial electrical instability and promote pro-fibrotic signalling.

**Conclusion:**

Our results identify atrial electrophysiological abnormalities and profibrotic remodelling as key mechanisms by which TTNtv increase AF risk, even in the absence of overt DCM. These findings support a primary atrial contribution to TTNtv-associated arrhythmogenesis and provide mechanistic insight into AF as an early clinical manifestation in carriers.

## Graphical Abstract

**Graphical Abstract:**
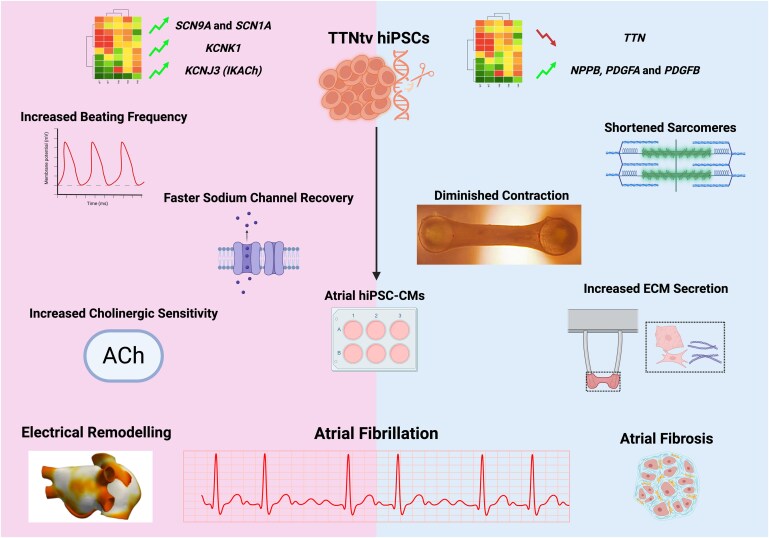

**Time of primary review: 28 days**

## 1. Introduction

Atrial Fibrillation (AF) is the most common cardiac arrhythmia, significantly increasing morbidity and mortality risk due to complications such as stroke, heart failure, dementia, and sudden cardiac death.^1–7^ AF is a supraventricular arrhythmia characterized by uncoordinated atrial activation resulting in ineffective atrial contraction with common risk factors including obesity, diabetes, heart disease and hypertension.^8,9^ In individuals >65 years old, AF prevalence exceeds 10%.^10^ Despite this, its underlying pathophysiological mechanisms remain poorly understood. In addition, AF can have a hereditary component,^11–13^ driven by both common and rare genetic variants. Rare genetic variants associated with early-onset AF, which can be characterized as the development of AF before the age of 66,^12^ are clustered in genes encoding constitutive parts of the cardiomyocyte contractile apparatus.^14–17^ Recently, a clear link has emerged between new onset AF and truncating variants of the giant sarcomeric protein titin (TTNtv).^17,18^ TTNtv increase the burden of arrhythmias in patients with dilated cardiomyopathy (DCM).^18–20^ Furthermore, TTNtv associated DCM often first clinically manifests with atrial arrhythmias.^21,22^ However, the precise mechanism(s) linking TTNtv with increased AF risk are unknown.

The founder TTNtv, *TTN* c.59926+1 G>A (rs553526525), described in Hoorntje *et al*.^23^ was identified in 11 probands. Retrospective analysis of *TTN* c.59926+1 G>A probands and family members revealed that 43% of carriers who developed DCM had early-onset AF (before the age of 66). In approximately 30% of cases, AF preceded the development of DCM, suggesting that in the presence of a cardiomyopathy-associated mutation, AF may reflect primary atrial myopathy rather than being a consequence of altered ventricular mechanics. Furthermore, early onset AF (as early as 34 years) occurred in 10% of *TTN* c.59926+1 G>A family members that did not develop DCM. This argues that specific mechanisms intrinsic to the atria may predispose to AF in the presence of TTNtv. Therefore, we set out to identify proarrhythmic mechanisms by generating an atrial human induced pluripotent stem cell-derived atrial cardiomyocyte (hiPSC-CM) model for this TTNtv.

The causative links between TTNtv and AF are poorly understood, with the majority of previous studies focusing on the pathophysiological effects variants have on DCM in ventricular hiPSC-CMs.^24^ Using CRISPR/Cas9 engineering, we created disease relevant atrial hiPSC-CMs carrying the titin truncating variant *TTN* c.59926+1 G>A to gain novel insights into the pathogenicity of this variant in both DCM and AF.

In atrial hiPSC-CMs, the TTNtv caused increased spontaneous action potential firing and time-dependent sodium channel recovery and greater action potential duration (APD) shortening in response to cholinergic stimulation. *In-silico* simulations, conducted in a detailed 3D human atrial model, indicate that a TTNtv can create a heterogeneous repolarisation substrate that is sufficient to initiate AF, even in the absence of structural remodelling (myocardial fibrosis) or ectopic triggers.

Moreover, this particular TTNtv impeded sarcomere formation and increased expression of pro-fibrotic stress markers. Engineered heart tissues, containing co-cultured TTNtv atrial hiPSC-CMs with wild type cardiac fibroblasts, demonstrated reduced contractile function and a significant increase in extracellular matrix protein secretion. This suggests that TTNtv confers an increased risk to fibrotic remodelling of the atria, which is a common feature of AF.

In conclusion, we have identified two pro-arrhythmic mechanisms by which TTNtv is likely to increase the risk of AF.

## 2. Methods

### 2.1 CRISPR/Cas9-mediated generation of TTNtv hiPSCs

The KOLF2-C1 (WTSIi018-B-1) hiPSC line was used for all experiments in this study and was provided by the Wellcome Sanger Institute. The reconstituted ribonucleoprotein complex of 1 μL of 1 μg/μL guide RNA containing *TTN* c.59926+1 targeting crRNA (5′-AGAGAAAGCAACUUACGGAG −3′; IDT) and 0.6 μL of 20 μM Alt-R® S.p. HiFi Cas9 Nuclease (IDT) was electroporated into hiPSCs in the presence of a HDR donor carrying *TTN* c.59926+1 G>A (TTNtv) and *TTN* c.59910 G>A (silent *Hin*dIII site). Isolated colonies of the electroporated cells were established and genotyped by genomic PCR using primers encompassing both of the modification sites (F: 5′-AACCTGGGCCACCTAGAGAT-3′, R: 5′-GGAGACCAAGACCCACAATG-3′, 637-bp product) (see Supplementary material online, *Figure S1*). Specific *Hin*dIII digestion patterns of the PCR amplicons (280 and 357 bp) allowed identification of integration events of the HDR donor, which was subsequently verified by Sanger DNA sequencing. Successful colonies were validated as pluripotent and normal hiPSCs by FACS analysis for expression of stem cell markers including OCT4, SOX2, NANOG and SSEA4 using Human and Mouse Pluripotent Stem Cell Analysis Kit (BD Biosciences, Supplementary material online, *Figure S2*) and karyotyping by chromosomal counting at metaphase (see Supplementary material online, *Figure S3*) performed by the Chromosome Dynamics Core Facility at the Wellcome Centre for Human Genetics, UK. Heterozygous (Het) and homozygous (Hom) TTNtv hiPSC lines were established, and their genotypes were verified through analysis of the integrated *Hin*dIII restriction enzyme sites in the guide RNA and confirmed by sequencing (see Supplementary material online, *Figure S4*).

### 2.2 hiPSC culture and differentiation into atrial cardiomyocytes

hiPSCs were cultured and differentiated into atrial hiPSC-CMs as previously described in.^25^ Briefly, hiPSCs were cultured in mTeSR Plus medium under humidified conditions at 37°C and 5% CO_2_. Cells were passaged at 70–80% confluency onto Geltrex®-coated 6-well plates, in mTeSR Plus medium supplemented with 10 μM of the ROCK inhibitor Y-27632. hiPSCs were differentiated into cardiac progenitor cells through activation of the WNT signalling pathway using 10 ng/mL BMP-4 and 8 ng/mL Activin A.

WNT inhibition and cardiomyocyte derivation was achieved through the addition of 10 μM XAV 939 and 10 μM KY 02111. Atrial specification was achieved through the addition of 1 μM retinoic acid on days 4 and 6 of differentiation. Cardiomyocyte metabolic selection was carried out through glucose starvation for 48 h, 12 days after the initiation of differentiation. The hiPSC-CMs were dissociated by washing once with PBS and incubating in TrypLE™ Select Enzyme (10X), no phenol red (ThermoFisher Scientific, A1217702) for 30 min. Cells were washed off with pre-warmed DMEM/F-12 (ThermoFisher Scientific, 11320033) and centrifuged for 10 min at 100 xg. Cells were counted using a haemocytometer and seeded at an appropriate cell density.

### 2.3 Atrial/ventricular hiPSC-CM analysis

Analysis on the successful generation of atrial hiPSC-CMs was performed by assessing the presence of structural, genetic and electrophysiological markers. Action potentials were recorded from a single batch of atrial (1 μM retinoic acid on days 4 and 6 of differentiation) and ventricular hiPSC-CMs. The stimulated action potentials recorded from atrial hiPSC-CMs showed reduced upstroke velocities, shortened APDs and more atrial-like action potential morphologies (APD (30–40)/(APD70–80)). The APD morphology is calculated using APD (30–40)/APD (70–80), with a value >1.5 indicating an atrial like AP.^26^ Atrial hiPSC-CM showed up-regulation of the atrial-specific ion channel subunit gene *KCNJ3*, as well as the atrial specific isoform of the myosin heavy chain (*MYH6*). Localized expression of the ventricular/atrial specific isoforms of the myosin light chain (MLC) were analysed by immunofluorescence. Atrial and ventricular hiPSC-CMs were stained for both MLC-2a (atrial) and MLC-2v (ventricular). Images were acquired at random and the number of cells positive for each or both of the markers was counted in each image. Compartment specific assessment of the atrial and ventricular hiPSC-CMs is shown in Supplementary material online, *Figure S5*.

### 2.4 Engineered heart tissues

#### 2.4.1 Generation and culture of hiPSC-derived engineered heart tissues

Atrial engineered heart tissues (EHTs) were generated using hiPSC-derived cardiac fibroblasts (hiPSC-CFs) and hiPSC-CMs as detailed in Cumberland *et al*.^25^ In brief, hiPSC-CFs were differentiated from KOLF2-C1 hiPSCs using a protocol adapted from Zhang *et al*.^27^ and retained in a quiescent state through the addition of 10 μM of the TGF-β1 inhibitor SB431542. Day 15 atrial hiPSC-CMs were combined with hiPSC-CFs (1 × 10^6^ hiPSC-CMs and 1.5 × 10^5^ hiPSC-CFs) to form a co-cultured EHT. The EHTs were cultured until 30 days after the initiation of differentiation in media containing 10 μM of the TGF-β1 inhibitor SB431542 to prevent chronic activation.^28^ The TGF-β1 inhibitor was removed on day 30 to assess activation over a defined period. Tissue/supernatant was harvested on day 35 (5 days without the TGF-β1 inhibitor).

#### 2.4.2 Contractility analysis of engineered heart tissues

Automated pixel tracking and signal analysis was utilized for the determination and analysis of EHT contractile variables. EHTs were recorded at day 35 for up to 30 s at 60 frames per second, using either a mobile phone (Apple iPhone 12) or a compact action camera (Yolansin C16). Videos were cropped to the central area around the EHT and re-encoded using FFmpeg (v7.0.2), prior to tracking the innermost edges of each EHT’s flexible support poles using the online mode of CoTracker3^29^ via a bespoke script. The pixel distance between poles over time was calculated to generate a beat curve for each EHT. Beat curves were smoothed using a Savitzky-Golay filter for downstream analysis using peak finding algorithms with the SciPy signal processing subpackage (v1.14.1). Peaks (beats) were automatically detected with a minimum prominence of 2.3 to prevent erroneous peak detection and a relative height of 0.5 was used to calculate peak width (i.e. full width at half maximum). The real-world scale (pixels per mm) for each EHT was determined by measuring the pixel diameter of one support pole of known size from each batch of videos. Contraction force (mN) was determined by converting beat amplitude (minimum inter-pole distance; mm) using a conversion factor of 0.255663465, calculated using the equation:


$$\mathrm{Force}=3A\pi Er^{4}/(64L^{4})$$

where *A* is beat amplitude, *E* is Young’s modulus of 3.0 MPa (measured using force callipers), *r* is pole radius (1 mm), and *L* is pole length (12 mm).

Beat amplitude, contractile force, beat duration (ms), contraction duration (ms), relaxation duration (ms), and peak-to-peak duration (s) were determined for each detected beat and beats per minute (BPM) and maximum relaxation (maximum inter-pole distance; mm) were calculated for each beat curve. The entirety of each video was utilized for analysis when possible; the EHT beat curve was trimmed to avoid starting or ending analysis during a beat. Tracking quality was assured through the visual assessment of videos with overlaid tracking coordinates and accurate beat detection was confirmed by assessing automatically labelled beat curves. One video was excluded from analysis due to poor tracking, related to suboptimal video quality.

### 2.5 RT-qPCR

RNA was harvested from 2D hiPSC-CM samples at 30 days after the initiation of differentiation. RNA was harvested from EHTs at 35 days after the initiation of differentiation. RNA extraction was performed using the Direct-zol RNA Miniprep Kit (Zymo Research, R2050) according to the manufacturer’s protocol. cDNA was generated using the High-Capacity cDNA Reverse Transcription Kit (ThermoFisher Scientific, 4368814), according to the manufacturer’s protocol. RT-qPCR was performed in TaqMan™ Fast Advanced Master Mix (ThermoFisher Scientific, 4444557) using Taqman probes listed in Supplementary material online, *Table S1* or PowerUp™ SYBR™ Green Master Mix (ThermoFisher Scientific, A25741) using primers listed in Supplementary material online, *Table S2*. All RT-qPCR reactions were performed according to the manufacturer’s protocol and expression values were normalized to the housekeeping gene *GAPDH*. 10 ng of cDNA was used per reaction. Cycle threshold values were obtained from 3 technical replicates of each sample. Relative gene expression was calculated using the comparative cycle threshold method.^30^

### 2.6 Bulk RNA-Seq

#### 2.6.1 Sample preparation and sequencing

Five batches of atrial hiPSC-CMs were differentiated from WT and Het hiPSCs in tandem. RNA was isolated using the Direct-zol RNA Miniprep Kit (Zymo Research, R2050) according to the manufacturer’s protocol. RNA quality was measured using a 4150 TapeStation System. Samples with RNA Index numbers > 9.2 were subsequently used for bulk RNA sequencing. Library preparation was performed at Novogene as follows: mRNA was purified from total RNA using poly-T oligo-attached magnetic beads. Following fragmentation, the first cDNA strand was synthesized using random hexamers, whereas for the second cDNA strand dUTP was used for directional library and dTTP for non-directional library synthesis.^31^ The non-directional library involved end repair, A-tailing, adapter ligation, size selection, amplification, and purification (Novogene). The directional library, required end repair, A-tailing, adapter ligation, size selection, USER enzyme digestion, amplification, and purification (Novogene). Quality control for the library preparation included Qubit and real-time PCR for quantification and bioanalyzer for size distribution detection. Quantified libraries were pooled and sequenced in paired-end mode (read length 150nt) using the Illumina HiSeq 4000 (Novogene). Raw reads in fatsq format were processed using custom pipelines (Novogene) to facilitate trimming of reads with low quality nucleotides, reads with adapter contamination and reads with uncertain nucleotides constituting more than 10% of either read. Clean reads were aligned to the reference GRCh37 genome using Hisat2 v2.0.5.^32^

#### 2.6.2 Bioinformatic analysis of RNA-Seq data

Genes with low expression values (< 1 count per million) were filtered out, and normalization factors for remaining genes were calculated with ‘DGEList’, using the trimmed means of m (TMM) method.^33^ Voom transformation^34^ was applied for normalization and a statistical model was fit with lmFit,^35^ using batch as a covariate in the model. The ebayes function was used to calculate moderated test statistics.

The topTable function was using to generate logFC and *P*-values, and adjusted *P*-values with Benjamini-Hochberg correction. Genes with logFC >1.5 and adjusted *P*-value <0.1 were considered differentially expressed. The normalized expression values were then batch corrected with the removeBatchEffect function from limma and principal component analysis was used via prcomp to assess sample clustering. Gene set enrichment analysis (GSEA) was performed with the gene ontology (GO) gene set using clusterProfiler.^36^ Adjusted *P*-values for GSEA were calculated with FDR correction, and pathways were considered significant with an adjusted *P*-value <0.05.

### 2.7 ELISA and titin gel

#### 2.7.1 ELISA

Enzyme-linked immunosorbent assays (ELISAs) were performed using cell culture supernatant. ELISAs performed on 2D hiPSC-CMs utilized cell culture media harvested from cells 30 days after the initiation of differentiation. ELISAs performed on EHTs were done using supernatant harvested 35 days after the initiation of differentiation (5 days after removal of the TGF-β1 inhibitor, SB431542). All ELISAs were performed according to the manufacturer’s protocol. The ELISAs used in the study are listed in Supplementary material online, *Table S3*.

#### 2.7.2 Titin gels

Titin gels were performed as described in Smart *et al*.^37^ Briefly, cell pellets were homogenized in sample buffer containing 8 M urea, 2 M thiourea, 3% SDS, 75 mM DTT, 10% glycerol, bromophenol blue and 0.05 M Tris·HCl, pH 6.8. Samples were heated in a water bath at 60°C for 30 s and then incubated at room temperature for 2 min (both while using a glass-glass homogenizer to shear every 15 s). Then the samples were incubated for 5 min on ice and cleared by centrifugation (15 000 *g*, 5 min). Agarose-strengthened SDS-PAGE (2% polyacrylamide; 0.5% agarose) was performed, at 2 mA overnight, and protein bands were visualized by SYPRO-Ruby (Molecular Probes, cat. no. S12000), according to the manufacturer’s instructions, and visualized on the Gel Doc EZ System.

### 2.8 Immunofluorescence

At day 26 after the initiation of differentiation, hiPSC-CMs were dissociated and passaged onto 10 mm geltrex coated coverslips. Four days later, the cells were fixed in 4% PFA in PBS for 14 min at room temperature. Samples were blocked for 1 h at room temperature in a blocking buffer consisting of 5% Foetal bovine serum, 1% Bovine Serum Albumin (Merck Millipore, A9418–10G) and 0.5% Triton™ X-100 (Merck Millipore, X100–100ML) in PBS. Primary antibodies were added in blocking buffer for 2 h at room temperature at concentrations detailed in Supplementary material online, *Table S4*. Samples were washed 3 times in PBS prior to the addition of secondary antibodies for 1 h at room temperature (covered from light) and at the concentrations detailed in Supplementary material online, *Table S4*. The nuclei stain DAPI was added with all secondary antibodies at the concentration detailed in Supplementary material online, *Table S4*. Coverslips were washed 3 times in PBS prior to mounting onto cover slides using Hydromount Histology Mounting Media (Scientific Laboratory Supplies, NAT1324). Images were acquired using a Zeiss LSM780 Confocal laser scanning microscope equipped with a C-Apochromat 63×/1.20 W oil objective.

### 2.9 Sarcomere analysis

Sarcomere analysis was performed on images acquired from atrial hiPSC-CMs 30 days after the initiation of differentiation, stained for α-actinin and phalloidin (F-Actin). Images were acquired from 3 differentiation batches of WT and Het hiPSC-CMs. Images were converted into tifs and analysed using the automated z-disc mapping software described in.^38^ Mean sarcomere length, Z-disc/Actin myofilament orientation order parameter and continuous Z-line length were plotted for each genotype. Sarcomere length was calculated as the centre-to-centre distance between adjacent Z-discs, identified by α-actinin staining. Z-disc positions were automatically detected along individual myofibrils and inter–Z-disc distances were measured along the local myofibril axis and averaged per image. As cells were not treated with a pharmacological relaxing agent prior to fixation, the calculated sarcomere lengths likely reflect a population of cardiomyocytes fixed across a range of contractile states.

### 2.10 Whole-cell patch clamp electrophysiology of atrial hiPSC-CMs

#### 2.10.1 Cardiomyocyte isolation

At 27 days after the initiation of differentiation, atrial hiPSC-CMs were dissociated from 6 well plates through treatment with TrypLE™ Select for 30 min at 37°C and passage into wells of a 24 well plate containing 10 mm coverslips coated according to the manufacturers protocol in Geltrex. 4 × 10^4^ hiPSC-CMs were seeded into each well of a 24 well plate. The cells were cultured in the 24 well plate for a further 3 days prior to patch clamp analysis. Coverslips were transferred into a recording chamber and continually superfused with extracellular solution at 3 mL/min^−1^.

#### 2.10.2 Patch-clamp recordings

Patch-clamp recordings and analysis were performed using an Axopatch 200B amplifier (Molecular Devices, USA). Analog signals were digitized at 50 kHz using a CED micro1401 operated using Signal v6 software (Cambridge Electronic Design, Cambridge, UK).

#### 2.10.3 Voltage clamp solutions

Whole cell patch clamp recordings were performed for outward potassium (I_K_) and inward sodium current (I_Na_). I_K_ was recorded at 37°C. The internal solution contained (in mM): KCl 135, NaCl 5, EGTA 10, HEPES 10, MgATP 3, Na_3_GTP 0.5 and D-glucose 10, pH of 7.2 (adjusted using KOH). The extracellular solution contained (in mM): NaCl 140, KCl 4.5, HEPES 10, D-glucose 10, NiCl 2, MgCl_2_ 1.2 and CaCl_2_ 1, pH of 7.4 (adjusted with NaOH).

I_Na_ was recorded at 37°C using an internal solution with a final pH of 7.2 (adjusted with CsOH). The solution contained (in mM): CsCl 115, HEPES 10, EGTA 10, MgATP 5, Tetraethylammonium Chloride (TEACl) 20, MgCl_2_ 0.5 and NaCl 5. The extracellular solution used in I_Na_ recordings had a final pH of 7.4 (adjusted with CsOH). The solution contained (in mM): NaCl 40, KCl 4.5, Choline Chloride (C_5_H_14_CINO) 100, HEPES 10, D-glucose 10, NiCl_2_ 2, MgCl_2_ 1.2 and CaCl_2_ 1.8. The low sodium solution used in these experiments allowed for sufficient voltage control.

#### 2.10.4 Voltage protocols

Whole-Cell Peak I_Na_ was elicited from a −100 mV holding potential using 100 ms pulses (100 to +40 mV in 5 mV steps). Steady state inactivation kinetics were assessed using 500 ms pre-pulses from −120 to −40 mV in 5 mV steps, followed by a 50 ms pulse to a test potential of −30 mV. Time-dependent recovery kinetics was assessed using a standard two pulse protocol (−100 mV to −30 mV, 25 ms). The time between the pulses incrementally increased between 1 and 900 ms. Peak I_K_ was evoked from a −70 mV holding potential using 500 ms pulses to test potentials −70 to +70 mV, in 10 mV increments.

#### 2.10.5 Action potential measurements in atrial hiPSC-CMs

Spontaneous and stimulated action potentials were recorded from atrial hiPSC-CMs at 30 days after the initiation of differentiation. Action potentials were recorded at 37°C using an internal solution of pH 7.2 (adjusted using KOH). The solution contained (in mM): KCl 135, NaCl 10, MgATP 5, HEPES 10, EGTA 0.1. Extracellular solution contained (in mM): NaCl 145, KCl 5.4, MgSO_4_.7H_2_O 0.83, NaH_2_PO_4_ 0.33, HEPES 5, Glucose 11, CaCl_2_ 1.8. Spontaneous action potentials were recorded from cells following a 60 s period after breakthrough, to allow cell stabilization. Ten successive spontaneous action potentials were analysed using custom algorithms developed in MatLab. A hyperpolarising current was then applied to cells to hold the diastolic membrane potential at—75 mV prior to recording of stimulated action potentials.^39,40^ Only cells that required a hyperpolarising current of < 150 pA were used.^39^ Action potentials were stimulated using a 1 nA, 2 ms current injection at a frequency of 1 Hz. 10 Successive stimulated action potentials were analysed from each cell.

#### 2.10.6 Carbachol treated action potential recordings

Spontaneous action potentials were recorded for 45 s prior to treatment of the cells with the extracellular solution listed above containing 2 μM carbachol for 2 min. Spontaneous action potentials were subsequently recorded, and the extracellular solution was switched back to one without carbachol. Action potentials were recorded following a further 2 min without carbachol to ensure that any changes seen in AP were due to carbachol treatment and not due to loss of seal/cell viability.

#### 2.10.7 Statistical analysis

Most statistical analyses were performed using GraphPad Prism (v10.0.03). Gaussian distribution was tested using a Shapiro-Wilk normality test. Normally distributed data were subjected to Welch’s *t*-test, non-normally distributed data were subjected to Mann–Whitney *U* test.

Statistical analyses for EHT variables were performed using R (v4.4.2) in RStudio (v2024.09.1). Intergroup differences for individual beat variables were determined using linear mixed models, with EHT ID specified as the random model to account for repeated measures, whereas simple linear models were used for aggregate beat curve variables. Model diagnostics included residual checks for homoscedasticity and normality.

#### 2.10.8 Optical mapping of calcium transients

WT and Het Atrial hiPSC-CMs were plated at a density of 2 × 10^6^ cells per 35 mm dish for a dense monolayer. hiPSC-CMs were imaged between days 25 and 30. For interrogation of calcium transients all experiments were performed as described in^41^ and.^42^ Briefly, cells were incubated with 10 μM Fura-2, AM (Invitrogen™, F1221) in 1 mL RPMI 1640 + B27 Supplement for 20 min at 37°C. Cells were then incubated with Tyrode’s solution for a further 20 min at 37°C to allow the dye to de-esterify and the cells to acclimatize to the Tyrode’s solution. The solution contained (in mM): NaCl 130, KCl 5.4, HEPES 14.1, Glucose 10, CaCl_2_ 1.8 and MgCl2 1.2. Fura-2, AM dye was excited at 380 nM with fluorescence passing through a 510 ±40 nm emission filter. Spontaneous calcium transients were recorded using an Evolve delta 512 × 512 EMCCD camera at a frame rate of 588 Hz with the WinFluor software. Data was analysed using Electromap.^43^

### 2.11 Computer modelling and simulation of human atrial electrophysiology

#### 2.11.1 *In-silico* human atrial CM models

An *in-silico* population of 1000 human atrial CM models was generated as in Muszkiewicz *et al*.^44^ to capture the electrophysiological variability reported in atrial hiPSC-CMs. The population was constructed with a modified version of the CRN model^45^ that included I_KACh_.^46^ Moreover, the I_KACh_ formulation was extended to consider I_KACh_ activation dependent on acetylcholine analog carbachol.^47^ The ionic current densities of the modified CRN model were sampled up to ±50% of their control ranges, using Latin Hypercube sampling. The resulting 1000 CM models underwent calibration against action potential biomarkers derived from human patients in sinus rhythm and AF.^48^ Thus, from the original population, 830 atrial CM models presented action potential biomarkers in range with the human data, and were subsequently used to analyse the effects of 2 μM carbachol, as conducted experimentally with atrial hiPSC-CMs.

#### 2.11.2 *In-silico* human whole-atria models

Two atrial CM models reproducing the experimental conditions for WT and Het TTNtv hiPSC-CMs were chosen to conduct multi-scale simulations, using the detailed whole-atria model described in Ferrer *et al*.^49^ The virtual human atria included a realistic location of ganglionated plexuses, allowing simulation of a heterogeneous acetylcholine release in the atria.^50^

The single-cell properties of the atrial CM model were included in the right atrial tissue and were scaled in the left atrium, crista terminalis, pectinate muscles, left atrial appendage and atrio-ventricular rings, according to reported regional differences on ion channel expression.^51^ Sinus rhythm simulations were conducted considering a maximum release of 0.1 μM acetylcholine in ganglionated plexus sites.^50^

## 3. Results

### 3.1 Generation of hiPSCs carrying the clinically relevant TTNtv

The TTNtv *TTN* c.59926+1 G>A variant described in Hoorntje *et al*.^23^ is a single nucleotide change located in the boundary of exon 303, which is part of the A-band section of titin. This variant is associated with a high incidence of early-onset atrial fibrillation (AF), with nearly half of affected patients presenting at an early age (<66 years old). To study mechanisms that predispose to AF, the genetic change was introduced into a healthy hiPSC line (WT) by CRISPR/Cas9 technology, generating heterozygous (Het) and homozygous (Hom) cells carrying the genetic variant (see Supplementary material online, *Figures S1*  to  *S4*). Pluripotency and karyotype integrity were confirmed (see Supplementary material online, *Figures S2* and *S3*).

### 3.2 Sarcomere integrity and TTN gene expression

**Figure 1:**
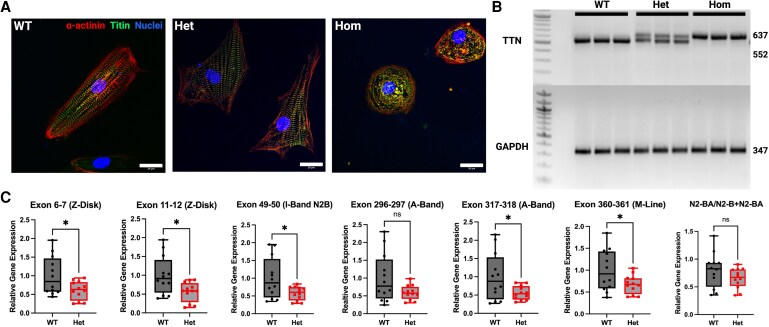
Differential sarcomere integrity and *TTN* gene expression in WT, het and hom atrial hiPSC-CMs. (*A*) hiPSC-CMs were stained for α-actinin (red), Titin (z-disc region, green) and DAPI (nuclei, blue). Scale bar = 20 μm. (*B*) RT-PCR was performed using primers that annealed at exons 303 and 304. The mutant *TTN* product can be distinguished from the WT allele (552 bp) by its larger amplicon (637 bp) (see Supplementary material online, *Figure S6*). (*C*) WT and Het atrial hiPSC-CMs were analysed for expression of *TTN* by qPCR using primer pairs that bind within different exons. Primers designed to amplify Exon 11–12 were specific for the N2-BA isoform of TTN. The primers designed to amplify Exon 49–50 were specific for both N2-B and N2-BA isoforms. N2-BA/N2-B+N2-BA correlates with the proportion of compliant TTN isoforms (*N* = 4 Differentiation Batches, *n* = 12 Technical Replicates). A Welch’s *t* test was used to ascertain significance (*denotes *P* < 0.05).

Atrial hiPSC-CMs were differentiated from the isogenic trio (WT, Het, Hom) (*Figure 1A*). The cells demonstrated chamber specific, i.e. atrial vs. ventricular, electrophysiological, transcriptional, and structural markers (see Supplementary material online, *Figure S5*). RT-PCR of the region between exons 303 and 304 of *TTN*, showed the expected product size of 552 bp for WT, 552 and 637 bp for Het and 637 bp for Hom atrial hiPSC-CMs (*Figure 1B*). Sequence analysis of the 637 bp PCR product indicated retention of the intron (85 bp) immediately downstream of the exon 303, which is predicted to contain a premature stop codon *TTN* p.H10911RfsX16 in the cardiac *N*-2B isoform of titin (see Supplementary material online, *Figure S6*).

Examining exon-specific gene expression is crucial when investigating TTNtv, as alternative splicing plays a significant role in determining the length of the translated protein; mutations in specific exons can disrupt splicing patterns, leading to variable impacts on titin’s structure and function^52^. *TTN* gene expression was broadly down-regulated across exons spanning the full length of the transcript in Het atrial hiPSC-CMs (*Figure 1C*). Total *TTN* transcript levels in Het cells were consistently reduced to approximately 50% of WT, indicating partial transcriptional silencing of the mutant allele. Despite this reduction, the N2-BA / (N2-B + N2-BA) isoform ratio was unchanged (*Figure 1C*), and titin gel analysis confirmed no significant difference in protein isoform expression between Het and WT cells (see Supplementary material online, *Figure S7*).

In contrast to WT and Het, which displayed functional and organized sarcomeres (*Figure 1A*), the Hom counterparts showed impaired sarcomere formation and consequently lacked contractile activity. As biallelic TTNtv are embryonic lethal,^52,53,55,56^ and hence rarely found in the clinic, the remainder of the study focused on WT and Het atrial hiPSC-CMs to explore pro-arrhythmic mechanisms.

### 3.3 Atrial hiPSC-CM sarcomere length and cardiac stress

**Figure 2:**
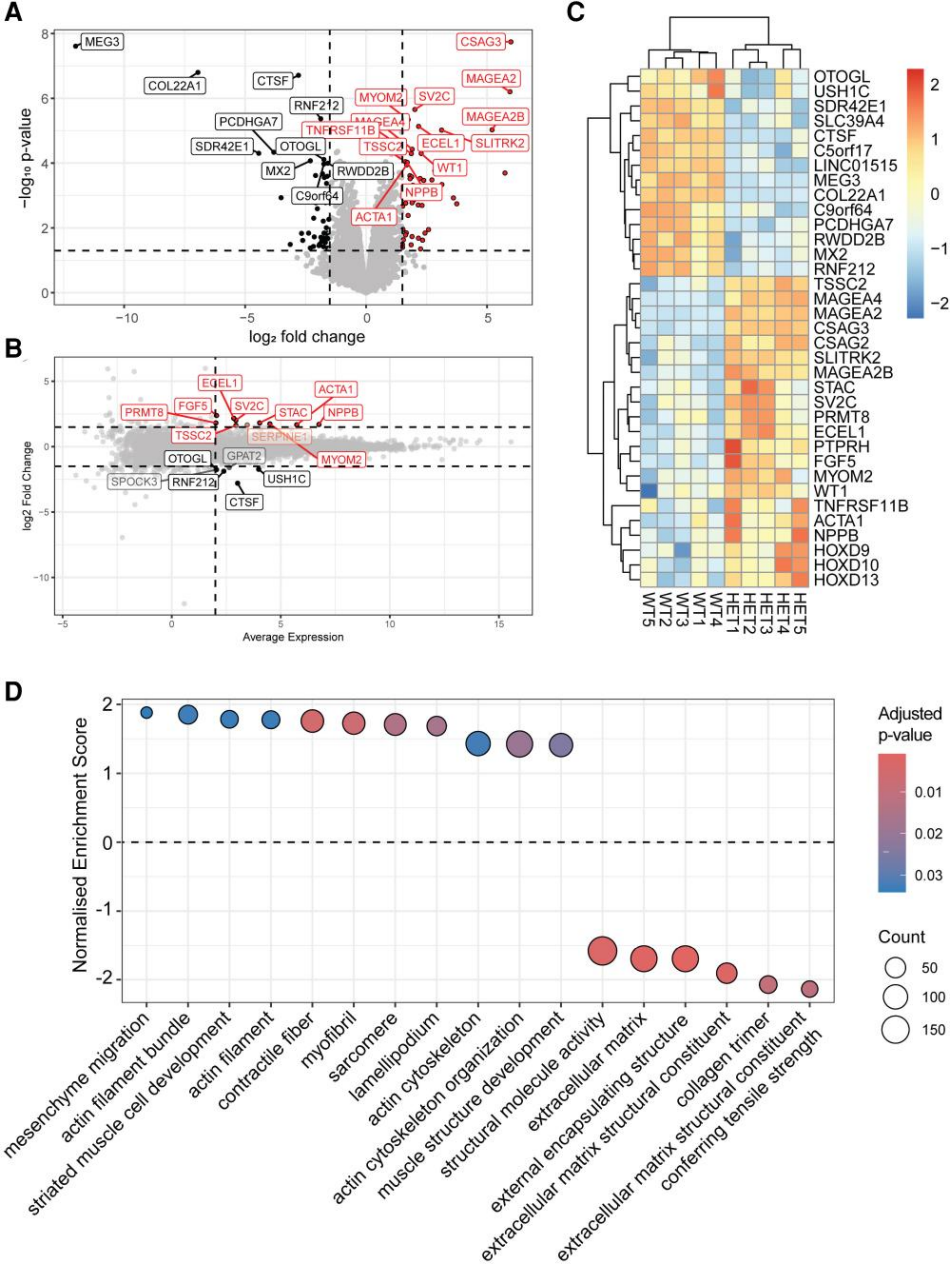
Bulk RNA-Seq of WT and het atrial hiPSC-CMs. (*A*) Volcano Plot of WT and Het hiPSC-CM Gene Expression. The *x*-axis represents the log_2_ fold change in gene expression between Het and WT hiPSC-CMs, while the *y*-axis displays the -log_10_  *P*-value for each gene. Genes with a log_2_ fold change > 1.5 and a *P*-value below 0.05 (denoted by dashed lines) are highlighted in either red (up-regulated in Het hiPSC-CMs) or black (down-regulated in Het hiPSC-CMs). (*B*) MA plot (average expression vs. log fold change) in WT and Het hiPSC-CMs. Genes with a log_2_ fold change > 1.5 and an average expression > 2 (denoted by dashed lines) are highlighted in either red (up-regulated in Het hiPSC-CMs) or black (down-regulated in Het hiPSC-CMs). (*C*) Heatmap of sample gene expression for 35 differentially expressed genes in WT and Het hiPSC-CMs. (*D*) Gene pathways which are up and down-regulated in Het hiPSC-CMs. Pathways are listed along the *x*-axis, while the *y*-axis represents the normalized enrichment score (NES), indicating the degree of enrichment for each pathway. Bubble size corresponds to the number of genes involved in each pathway, and the colour gradient represents the adjusted *P*-value, with red indicating more significant pathways (lower *P*-values).

No overt differences were observed in the structural morphology of atrial iPSC-CMs derived from WT and Het hiPSCs. To investigate unbiased changes in gene regulation that may contribute to the pathophysiology of the TTNtv, we performed bulk RNA-seq on WT and Het atrial hiPSC-CMs (*Figure 2*).

Transcriptomic profiling of Het atrial iPSC-CMs identified 70 differentially expressed genes (FDR <0.10). Core sarcomeric transcripts including *ACTA1* (α-actin), *MYOM2* (myomesin-2), *STAC1*, plus additional Z-disk regulators (*PDLIM3*, *FHL1*) were significantly up-regulated (*Figure 2A–C*), signalling a compensatory reinforcement of the contractile apparatus. In contrast, structural extracellular-matrix components such as *COL22A1* (collagen XXII), *VCAN* (versican) and *SPOCK3* were among the most strongly down-regulated, indicating an overall attenuation of matrix deposition and tensile strength. Cardiac stress markers showed a split pattern: *NPPB* (BNP) rose sharply, whereas the imprinted long non-coding RNA *MEG3* (and its locus-mate *MEG8*) was markedly suppressed. Gene-set enrichment analysis corroborated these findings, revealing robust enrichment of programs governing contractile fibre and myofibril organization in Het iPSC-CMs (*Figure 2D*), while pathways related to extracellular-matrix organization and collagen fibril formation were significantly depleted. Overall, an increase in *NPPB* and sarcomere-repair genes (*PDLIM3* and *FHL1*), alongside lower ECM and *MEG3* expression, suggests that the TTNtv cells may be under mechanical stress.

**Figure 3:**
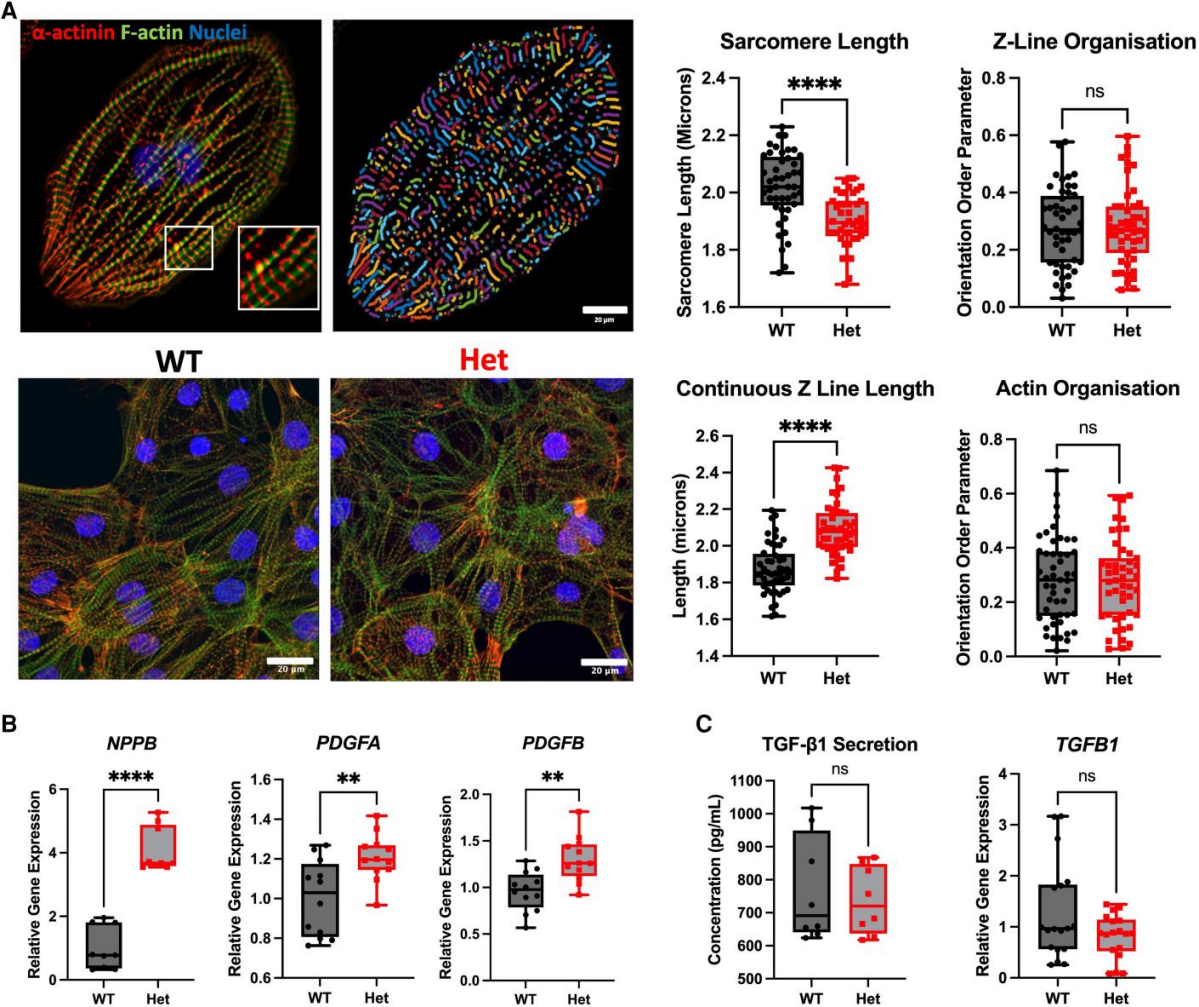
Sarcomere shortening and augmented cardiac stress in het atrial hiPSC-CMs. (*A*) Top Left: atrial hiPSC-CMs were stained for α-actinin, F-Actin and DAPI (Nuclei). Scale bar = 20 μm. Top Right: The Z-lines and thin myofilaments of the cells were mapped and analysed using a Z-line detection script^38^ to ascertain sarcomere length, continuous Z line length and Z line and Actin myofilament Organization (orientation order parameter). WT *N* = 3, *n* = 50, Het *N* = 3, *n* = 47. (*B*) RT-qPCR demonstrated a significant increase in *NPPB* (*N* = 3, *n* = 9), *PDGFA* and *PDGFB* (*N* = 4, *n* = 12) expression. (*C*) Gene (*N* = 6, *n* = 18) and protein expression (ELISA) (*N* = 4, *n* = 8) of the pro-fibrotic cytokine TGF-β1 was unchanged in the Het cells. A Welch’s *t* test was used to ascertain significance (**** denotes *P* < 0.0001, ** denotes *P* < 0.01, ns = *P* > 0.05).

To directly assess sarcomere organization and structure, day 30 atrial iPSC-CMs were fixed and stained for α-actinin, F-actin, and DAPI. Quantitative image analysis using a Z-line and thin myofilament detection algorithm^38^ demonstrated that while sarcomere alignment was preserved (*P* = 0.94), sarcomere length was significantly reduced in Het cells (*P* < 0.0001) relative to WT (*Figure 3A*). This was accompanied with an increase in mean continuous Z line length in the Het hiPSC-CMs but no significant difference in actin myofilament organization.

To assess whether the TTNtv induced early transcriptional changes associated with stress or remodelling, we examined the expression of pro-fibrotic genes involved in the activation of cardiac fibrosis (see Supplementary material online, *Figure S8*). Bulk RNA-seq revealed up-regulation of *NPPB*, a canonical marker of cardiomyocyte stress, which is strongly associated with the onset and progression of dilated cardiomyopathy.^57^ This was confirmed by RT-qPCR (*Figure 3B*). Although expression of the pro-fibrotic growth factors *PDGFA* and *PDGFB* were not significantly up-regulated in the bulk RNA-seq, qPCR analysis confirmed their increased expression in Het atrial iPSC-CMs (*Figure 3B*). Despite increased expression of pro-fibrotic genes in the Het atrial hiPSC-CMs, neither gene expression nor secreted levels of TGF-β1 were significantly altered, as assessed by qPCR and ELISA, respectively (*Figure 3C*). This suggests that while certain pro-fibrotic mediators are transcriptionally activated, a full fibrotic program is not established in a hiPSC-CM monoculture. As such, the observed changes likely reflect an early stress-associated paracrine signature rather than direct fibrotic transformation. These signals may play a priming role *in vivo*, where fibroblasts and non-myocytes are present to respond to such cues.

Taken together, these findings indicate that Het atrial hiPSC-CMs exhibit both transcriptional and structural alterations, including sarcomere shortening, Z line lengthening and selective activation of stress-related and pro-fibrotic mediators. However, the concurrent down-regulation of other fibrosis-associated genes, including *COL22A1* and *MEG3*, suggests that a full fibrotic program is not established. Instead, the data support an early stress-associated remodelling phenotype, which may contribute to the development of a pro-arrhythmic substrate in TTNtv-mediated atrial fibrillation and could evolve into a full fibrotic response in the presence of cardiac fibroblasts.

### 3.4 Assessing pro-fibrotic signalling in co-culture engineered heart tissues

Atrial fibrosis leads to the stiffening of the upper chambers of the heart, due to excessive deposition of extracellular matrix proteins by activated fibroblasts.^58^ It can disrupt normal electrical conduction and contribute to arrhythmia induction.^59^ Overexpression of *PDGFA* and *PDGFB*, as seen in the Het atrial hiPSC-CMs (*Figure 3B*), is linked to structural remodelling and cardiac fibrosis.^60–62^

**Figure 4:**
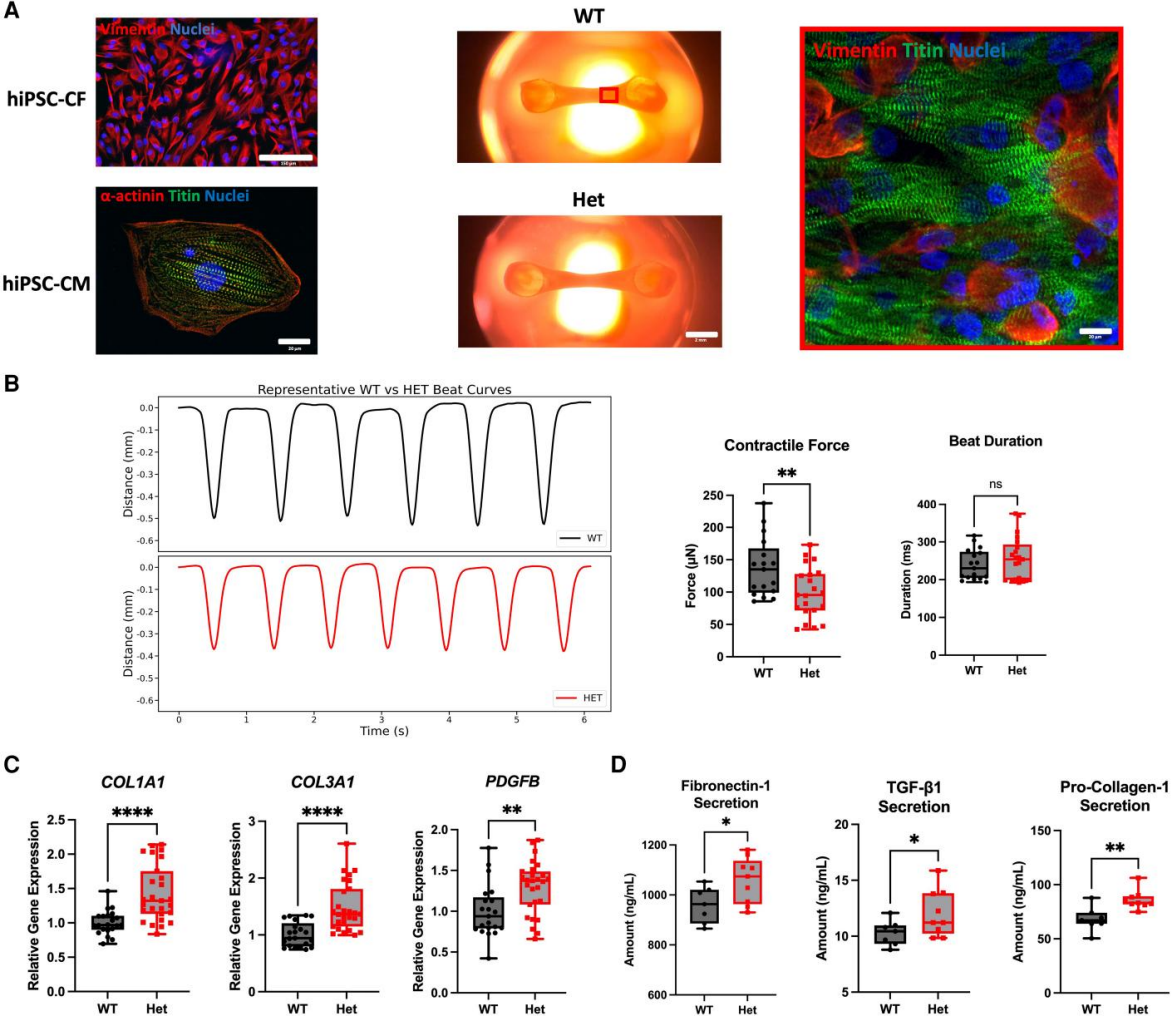
Pro-fibrotic gene expression and remodelling in Het EHTs. (*A*) Co-cultured EHTs constructed using atrial hiPSC-CMs and quiescent hiPSC-CFs (Scale Bars: hiPSC-CF = 150 μm, hiPSC-CMs = 20 μm, EHT = 2 mm, EHT IHC inlet = 20 μm). (*B*) Left: Representative beat curves showing patterns of contraction and relaxation of WT and Het EHTs. Right: Contractile Force and Beat Duration (WT *N* = 5 EHT batches, *n* = 17 EHTs; Het *N* = 5, *n* = 21). Linear mixed models were used to ascertain significance (** denotes *P* < 0.01). (*C*) qPCR was performed on EHTs for pro-fibrotic and extracellular matrix genes: *COL1A1*, *COL3A1* and *PDGFB* (*N* = 3 batches of EHTs, *n* = 7 EHTs for WT and *n* = 9 EHTs for Het, 3 technical replicates performed per EHT). A Mann–Whitney *U* test was used to ascertain significance for *COL1A1* and *COL3A1*. A Welch’s *t* test was used to ascertain significance of *PDGFB* (**denotes *P* < 0.01). (*D*) Supernatant was taken from the EHTs and ELISAs were performed for Fibronectin-1, TGF-β1and Pro-Collagen 1. A Mann–Whitney *U* test was used to ascertain significance for Fibronectin-1. Welch’s *t* test was used to ascertain significance for TGF-β1 and Pro-Collagen 1 (*denotes *P* < 0.05, **denotes *P* < 0.01, ****denotes *P* < 0.0001).

Co-cultured engineered heart tissues (EHTs) were established to assess the effect of the TTNtv on cellular crosstalk between cardiomyocytes and fibroblasts. EHTs were constructed using WT and Het atrial hiPSC-CMs and quiescent hiPSC-CFs (*Figure 4A*).^25^ The Het EHTs showed a reduction in contractile force, but no significant difference in beat duration or any other contractile properties (*Figure 4B*) (see Supplementary material online, *Figure S9*). The Het EHTs showed a significant up-regulation of the profibrotic and extracellular matrix genes: *PDGFB*, *COL1A1* and *COL3A1* (*Figure 4C*). ELISAs performed on the supernatant identified a significant increase in the secretion of the pro-fibrotic cytokine TGF-β1 and the extracellular matrix proteins: Pro-Collagen 1 and Fibronectin-1 in Het EHTs (*Figure 4D*). Overall, these findings suggest that Het TTNtv EHTs exhibit diminished contractile function and a heightened propensity for atrial fibrosis compared with their WT counterparts, highlighting its potential role in the development of a substrate for AF.

### 3.5 Electrophysiological analysis of atrial hiPSC-CMs

**Figure 5:**
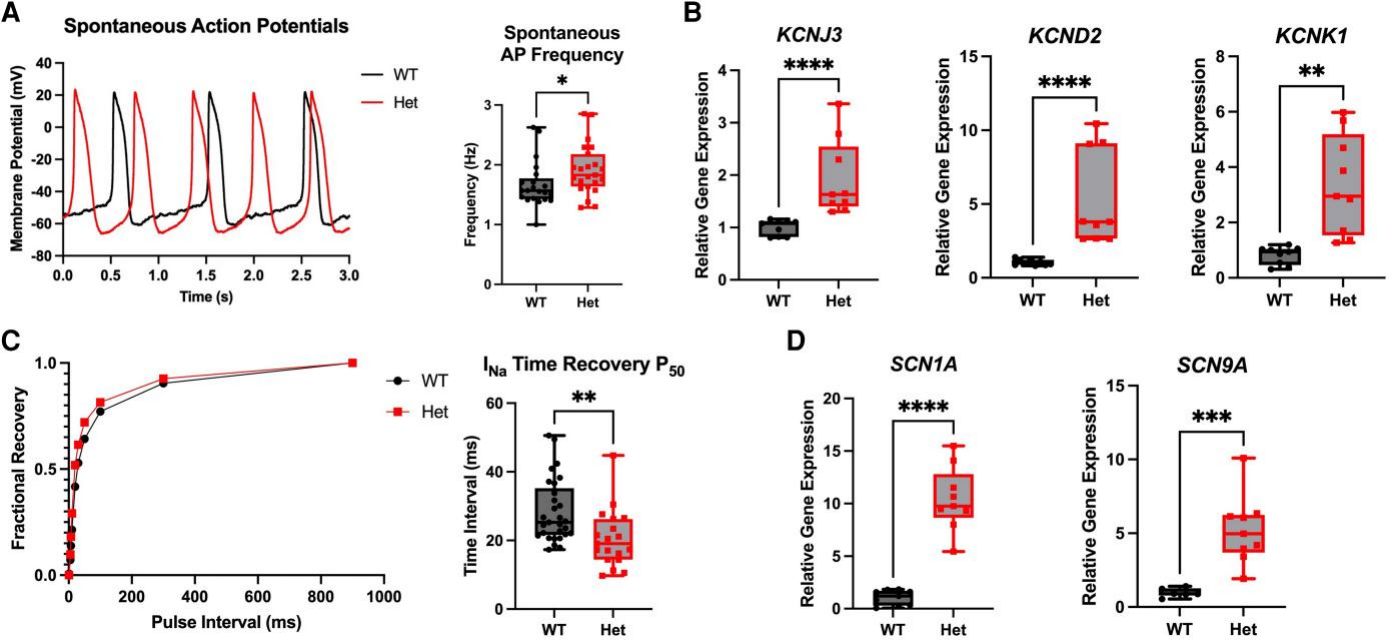
Alterations in spontaneous action potential frequency and ion channel subunit gene expression in WT and het atrial hiPSC-CMs. (*A*) Left: A representative spontaneous action potential trace from WT and Het atrial hiPSC-CMs. Right: Spontaneous action potential frequencies observed in WT (*N* = 3, *n* = 21) and Het hiPSC-CMs (*N* = 3, *n* = 23). A Mann–Whitney *U* test was used to ascertain significance (*denotes *P* < 0.05). (*B*) RT-qPCR was performed using primers designed to amplify ion channel subunit genes *KCNJ3*, *KCNK1* and *KCND2* (*N* = 3, *n* = 9). A Mann–Whitney *U* test was used to ascertain significance of *KCNJ3* and *KCND2* (****denotes *P* < 0.0001). A Welch’s *t* test was used to ascertain significance of *KCNK1* (**denotes *P* < 0.01). (*C*): Left: Time dependent recovery kinetics of I_Na_ Right: I_Na_ Time Recovery P_50_ (WT, *N* = 3, *n* = 33 and Het, *N* = 3, *n* = 19). A Mann–Whitney *U* test was used to ascertain significance (** denotes *P* < 0.01). (*D*) RT-qPCR was performed using primers designed to amplify ion channel subunit genes *SCN1A* and *SCN9A* (*N* = 3, *n* = 9). A Welch’s *t* test was used to ascertain significance (*denotes *P* < 0.05, **denotes *P* < 0.01, ***P < 0.001. *P* < 0.0001).

Electrical remodelling is fundamental in the initiation and progression of AF. Manual whole-cell patch-clamp recordings and optical calcium transient mapping were used to comprehensively evaluate how the TTNtv affects the intracellular electrophysiological properties of atrial hiPSC-CMs. Spontaneous and stimulated action potentials (AP) were recorded from WT and Het atrial hiPSC-CMs. Spontaneous action potential frequency was increased in Het cells, indicating increased automaticity (*Figure 5A*). qPCR analysis showed an increase in the expression of the ion channel subunit genes *KCNJ3*, *KCNK1* and *KCND2* in the Het cells (*Figure 5B*). Of these, *KCNJ3* has been linked with AF.^63^ Other major AF-associated ion channel genes such as *KCNA4, KCNA5, KCNE1, KCNJ2, KCNJ5, KCNJ8, CANA1C* and *SCN5A* were not differentially expressed between genotypes (see Supplementary material online, *Figure S10*). Furthermore, baseline AP morphology was not different between genotypes, including stimulated and spontaneous AP morphology (see Supplementary material online, *Figures S11* and *S12*). Measurements of peak voltage dependent I_Na_ (see Supplementary material online, *Figure S13*) and peak voltage dependent I_K_ (see Supplementary material online, *Figure S14*) were consistent between genotypes. Assessment of the biophysical parameters of sodium channel activity revealed no significant difference in the I_Na_ density, k activation, V_50_ inactivation and k inactivation (see Supplementary material online, *Table S5*).

Time-dependent sodium current recovery refers to the process by which voltage gated sodium channels return from an inactivated state (after an action potential) to a closed state (ready to open following a stimulus). Reduced sodium recovery time can cause a reduction in the effective refractory period at pathophysiologically high activation rates.^64^ Sodium current recovery (I_Na_ time recovery P50) was significantly faster in the Het cells (*Figure 5C*) and was accompanied by an up-regulation in the fast recovery sodium channel subunit gene *SCN9A* as well as *SCN1A* (*Figure 5D*). Optical mapping of calcium transients revealed no significant difference in intracellular calcium handling (see Supplementary material online, *Figure S15*). Overall, Het TTNtv hiPSC-CMs demonstrated increased beating frequencies, enhanced sodium current recovery and an up-regulation in the expression of the acetylcholine-sensitive K^+^ current subunit gene, *KCNJ3*.

### 3.6 Susceptibility of atrial hiPSC-CMs to acetylcholine receptor agonist carbachol

**Figure 6:**
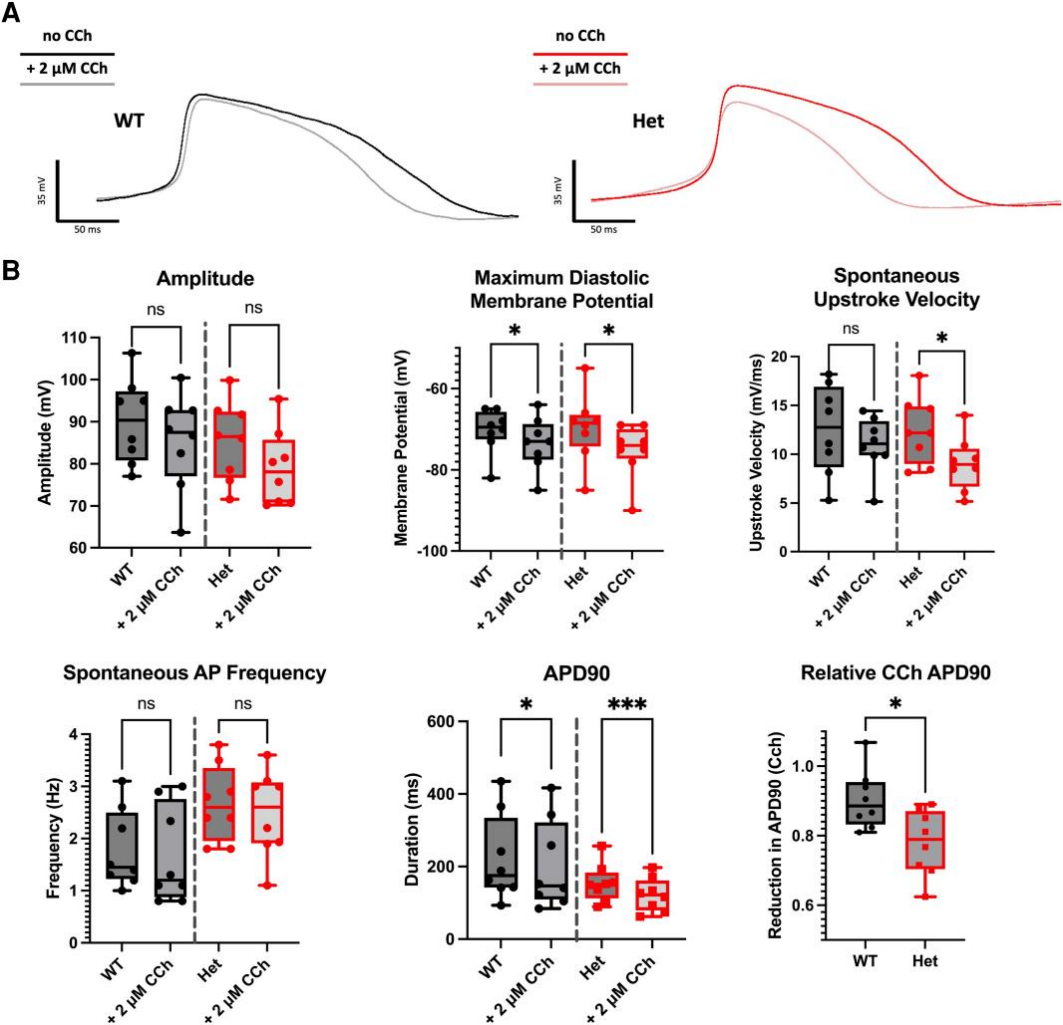
Enhanced electrophysiological sensitivity to carbachol in WT and het atrial hiPSC-CMs. Spontaneous action potentials (WT = black, Het = red) were recorded prior to and after the treatment of the cells with 2 μM of the acetylcholine receptor agonist carbachol (CCh) [in figure WT/Het label; control/carbachol]. (*A*) Representative traces of spontaneous action potentials recorded prior to and after the addition of 2 μM carbachol in WT and Het atrial hiPSC-CMs. (*B*) The effect of carbachol on AP amplitude, maximum diastolic membrane potential, spontaneous upstroke velocity, APD90 spontaneous AP frequency and relative APD90 post-carbachol treatment showing the mean and SD (*N* = 3, *n* = 8, *N* = 3, *n* = 8). A mixed effects one-way ANOVA was used to ascertain significance of carbachol treatment within each cell line (*P < 0.05, ***P < 0.001).

The ion channel subunit gene *KNCJ3* encodes the G protein-activated inward rectifier potassium channel 1 (GIRK1/K_ir_3.1), which combines with G protein-activated inward rectifier potassium channel 4 (GIRK4/K_ir_3.4) to regulate the parasympathetically activated acetylcholine-sensitive K^+^ current, *I*_KACh_ in sinoatrial and atrial CMs.^65^ Gain of function mutations in *KCNJ3*, which increase activity of GIRK1/4, have been linked to dysregulation in heart rate and an increased prevalence for AF.^63^ Here, spontaneous action potentials were recorded from WT and Het atrial hiPSC-CMs with and without 2 μM carbachol treatment (a I_KACh_ agonist). Carbachol was used instead of acetylcholine due to its greater stability and longer-lasting effects.^66^ Representative action potential traces from both genotypes in the presence and absence of carbachol are shown in *Figure 6A*. Treatment with carbachol caused a significant decrease in the upstroke velocity of the Het cells (*Figure 6B*). Furthermore, carbachol elicited a greater reduction in the duration of the APD in the Het hiPSC-CMs (22%) compared with the WT cells (10%) (*Figure 6A* and *B*). This demonstrates increased sensitivity to cholinergic stimulation in the TTNtv hiPSC-CMs. Carbachol treatment elicited no significant effect on the action potential amplitude or spontaneous AP frequency in WT or Het cells and caused a similar mild hyperpolarisation of the maximum diastolic membrane potential in both genotypes (*Figure 6B*).

### 3.7 Susceptibility of *in-silico* atrial CMs to acetylcholine receptor agonist carbachol

**Figure 7:**
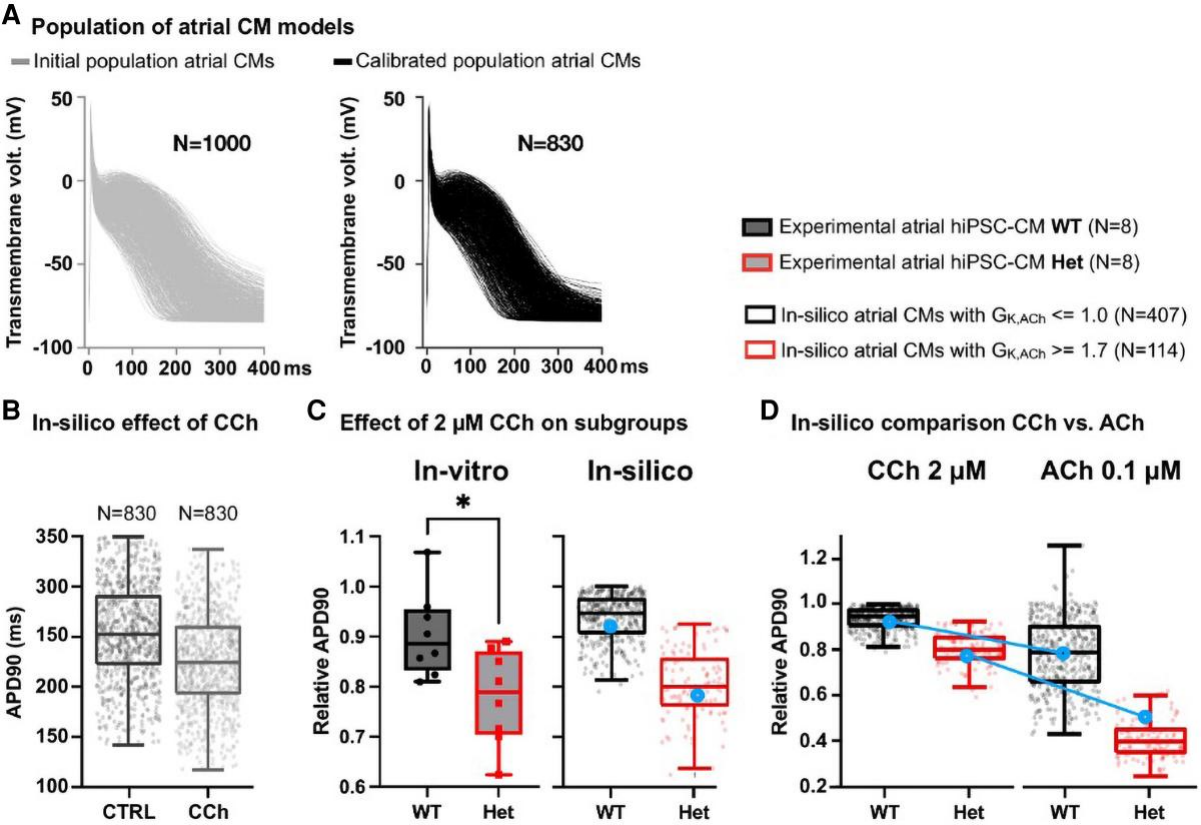
*In silico* action potential properties following carbachol and acetylcholine simulation. (*A*) Initial and calibrated population of atrial cardiomyocyte (CM) models. (*B*) APD at 90% of cellular repolarisation (APD90) for control conditions (CTRL) and after 2.0 μM carbachol (CCh) in the calibrated population of atrial CM models (*C*). Comparison of the relative APD90 (prior and after 2.0 μM CCh) *in-vitro* vs. *in-silico* for WT and Het atrial hiPSC-CMs (in-vitro, mixed effects one-way ANOVA, *P < 0.05 ) and atrial CM models with baseline (≤1, WT) or up-regulated (≥1.7, Het) I_KACh_ density (*in silico*) (*D*). *In-silico* comparison of the effects of 2.0 μM CCh vs. 0.1 μM acetylcholine (ACh) for the same subgroups shown in (*C*). The blue circle highlights the atrial CM model used for multi-scale simulations.

The sensitivity of atrial CMs to the acetylcholine receptor agonist carbachol was further analysed *in silico*. *Figure 7A* shows the action potential traces of the initial and calibrated population of human atrial CM models, and *Figure 7B* illustrates the effect of 2.0 μM carbachol had on the calibrated population. The calibrated population of 830 atrial CMs was split in two sub-groups: those representatives of WT and those of Het cells. Atrial CM models forming the WT subgroup presented a *I*_KACh_ density equal or lower than baseline (i.e. scaling factor ≤1). According to experimental data, the Het atrial hiPSC-CMs presented a 70% increase in *KNCJ3* relative gene expression compared with the WT cells. Therefore, the heterozygous subgroup included atrial CM models with a *I*_KACh_ density equal or higher than 70% of the WT cells (i.e. scaling factor ≥1.7). The *in-silico* subpopulation resembling the Het atrial hiPSC-CMs showed greater APD shortening occurring in response to 2.0 μM carbachol (*Figure 7C*). This enhanced sensitivity to cholinergic APD shortening was even more apparent when simulated in the presence of the carbachol analog, acetylcholine (ACh; 0.1 μM) (*Figure 7D*). The findings suggest that heterozygous atrial CMs exhibit a heightened sensitivity to cholinergic-induced APD shortening, likely due to increased I_KACh_ density, which may have important implications for understanding arrhythmogenic susceptibility.

### 3.8 TTNtv creates a heterogeneous repolarization substrate sufficient to precipitate AF in the *in-silico* human atria

**Figure 8:**
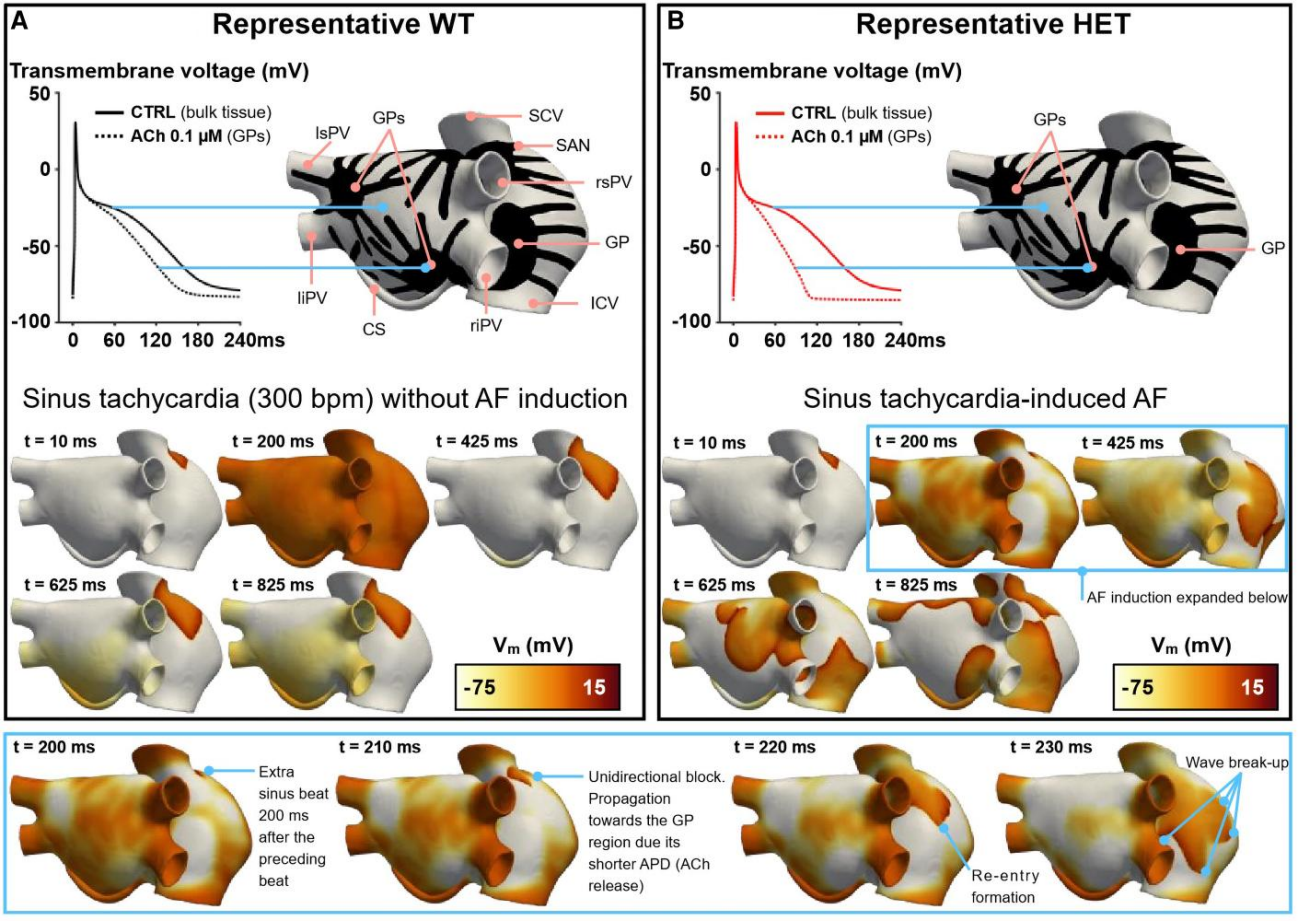
Sinus tachycardia-induced atrial fibrillation (AF) for WT and het conditions. (*A*) *In-silico* atrial cardiomyocyte model used for the WT, and a schematic representation of the human whole-atria model used for multi-scale simulations. Consecutive snapshots of the atrial transmembrane voltage (V_m_) show that under sinus tachycardia AF is not invoked. (*B*) *In-silico* atrial cardiomyocyte model used for Het TTNtv. Snapshots of V_m_ show AF induction and maintenance under sinus tachycardia. The initiation of AF for the Het condition is illustrated and explainedin the box at the bottom of the figure. SAN, sinoatrial node; SCV-ICV, superior and inferior cava vein; rs-ri-ls-li-PV, right superior, right inferior, left superior and left inferior pulmonary vein; CS, coronary sinus. The release of 0.1 μM acetylcholine only occurs at the ganglionated plexuses (GPs), arranged following an octopus configuration.

The two *in-silico* atrial CM models which best resembled the experimental data derived from the WT and Het atrial hiPSC-CMs were chosen for multi-scale simulations (*Figure 7C*). When these atrial CMs were exposed to 0.1 μM Ach, the relative APD90 decreased to 0.79 and 0.55 respectively for the WT and Het cells/models (*Figure 7D* and *Figure 8, top*). The ganglionated plexuses, which are clusters of nerve cells in the heart that release acetylcholine, play a crucial role in regulating heart rhythm. As expected, our simulations demonstrated that the release of 0.1 μM ACh from the ganglionated plexuses caused localized APD shortening and a heterogeneous repolarization substrate (see Supplementary material online, *Figure S16*). The heterogeneous repolarization was exaggerated in Het conditions, due to the up-regulation of I_KACh_ and consequently the greater APD differences between acetylcholine-release sites (i.e. ganglionated plexus) and bulk tissue (see Supplementary material online, *Figure S16* and *Figure 8*).

Atrial tachycardia, characterized by rapid but regular atrial beats, can trigger or precede AF, leading to irregular and disorganized atrial activity.^67^ The Het atrial hiPSC-CMs demonstrated a significantly higher spontaneous action potential frequency (*Figure 5A*), consistent with previous studies that have shown an increased prevalence of atrial tachycardia in patients with TTNtv-associated AF.^18,23^ In the event of sinus tachycardia (an extra beat applied 200 ms after the preceding beat), the heterogeneous repolarization substrate observed in the Het cell model was sufficient to evoke AF even in the absence of structural remodelling (*Figure 8B*). Under the same stimulation conditions, AF was not induced in the WT cell model (*Figure 8A*). This underscores how heightened cholinergic sensitivity may amplify trigger activity and promote the development of AF in patients with TTNtv.

## 4. Discussion

### 4.1 Key findings

Our study describes the effects of the familial AF TTNtv, *TTN* c.59926+1 G>A (rs553526525),^23^ in a newly generated atrial hiPSC-CM model. The TTNtv, located within a putative splice site at the end of exon 303, resulted in inclusion of the adjacent intronic sequence containing a premature stop codon. RT-qPCR revealed a broad down-regulation of *TTN* expression, with no truncated titin isoform being detectable via titin gel analysis. The variant caused dysregulation of genes associated with the structure and function of the contractile machinery, led to the formation of shortened sarcomeres and impeded contractile function in a 3D hiPSC-CM/CF model. The heterozygous hiPSC-CMs showed an up-regulation in cardiac stress markers and an increased propensity for cardiac fibrosis and structural remodelling, evidenced by increased expression and secretion of extracellular matrix proteins. Increased spontaneous beating frequencies and susceptibility to the acetylcholine receptor agonist carbachol, as well as increased time dependent sodium channel recovery indicate signs of pro-arrhythmic electrical remodelling. *In silico* simulations demonstrated that the observed disruption of cholinergic stimulation could facilitate the initiation of AF in human atria during sinus tachycardia.

### 4.2 Electrical remodelling in atrial hiPSC-CMs carrying a TTNtv

A thorough interrogation of the intracellular electrophysiology of WT and Het TTNtv hiPSC-CMs was performed. Although the majority of electrical functions were preserved in Het atrial hiPSC-CMs, we identified some important signs of electrical remodelling and changes in ion channel gene expression. In agreement with studies demonstrating that TTNtv confer increased sinus rates and a higher prevalence of atrial and ventricular tachycardia,^18,19,23,68^ we observed increased automaticity in the Het hiPSC-CMs (*Figure 5A*). Reduced time dependent recovery of inward sodium current was demonstrated in the Het cells (*Figure 5C*) and was accompanied by an up-regulation in the expression of sodium channel subunit genes: *SCN9A* and *SCN1A* (*Figure 5D*). Increases in time dependent sodium channel recovery may cause a reduction in effective refractory period at high activation frequencies, as seen during AF.^64^ Previous studies modelling TTNtv in hiPSC-CMs have shown an increase in *SCN9A* expression, highlighting this as a recurrent pathophysiological change.^69^ A significant up-regulation of the acetylcholine dependent potassium channel gene, *KCNJ3*, was observed in the Het hiPSC-CMs and is consistent with previous studies investigating TTNtv in ventricular hiPSC-CMs.^70^

Treatment of atrial hiPSC-CMs with the acetylcholine receptor agonist carbachol revealed an increased sensitivity in the Het hiPSC-CM, as evidenced by a significantly greater reduction in APD following treatment (*Figure 6*). Enhanced APD shortening in response to ACh release during cholinergic stimulation likely establishes a heterogeneous repolarisation substrate favoring AF in patients harboring this TTNtv, as demonstrated by our human *in silico* simulations (*Figure 8*). While these models clearly show that electrical remodelling alone can initiate AF, they were based on preserved atrial morphology and ganglionated plexus distribution as well as relative gene expression of *KCNJ3*, omitting structural remodelling processes such as dilation or fibrosis as well as the protein expression and localization of the ion channel.^18,52,71^

### 4.3 The *TTN* c.59926+1 G>A TTNtv causes an increased propensity for atrial fibrosis

Atrial dilatation and fibrosis are among the first symptoms to manifest in TTNtv animal models.^18,52^ Furthermore, recent studies have shown that DCM patients with TTNtv show greater left atrial dysfunction than non-carriers.^71^

This has prompted considerations as to whether the pathology of TTNtv can be described as an atrial cardiomyopathy.^72–74^ Previous studies modelling the effect of TTNtv in ventricular and atrial hiPSC-CMs have demonstrated cardiomyopathy-associated changes including sarcomere disorganization and contractile dysfunction.^69,70,75^ In this study, bulk RNA-seq revealed significant dysregulation of pathways associated with sarcomere formation, contractile machinery and extracellular matrix production (*Figure 2B*). Further targeted interrogation revealed a reduction in sarcomere length and the overexpression of the pro-fibrotic genes *NPPB*, *PDGFA* and *PDGFB* (*Figure 3A*). Changes in sarcomere length and organization have previously been demonstrated as a feature of TTNtvs in human adult CMs and ventricular hiPSC-CMs^75–78^.

Activation and overexpression of PDGF ligands and receptors is often induced by mechanical stress and is associated with the development of atrial fibrosis, AF and arrhythmogenic cardiomyopathies.^61,79–81^ Mechanical stress and tachypacing independently elicit increased paracrine mediated activation of cardiac fibroblasts through PDGF.^61,82^ Indeed, previous studies have linked *TTN* variants with up-regulated PDGF gene and protein expression and increased PDGF signalling.^81,83^

Co-cultured EHTs constructed from Het atrial hiPSC-CMs with cardiac fibroblasts exhibited decreased contractile function when compared with their WT counterparts (*Figure 4B*). This was likely due to aberrant expression of sarcomeric and contractile genes (*Figure 2*) and shortened sarcomere lengths (*Figure 3*), as evidenced by bulk RNA-seq and sarcomere analysis. Het EHTs showed significant up-regulation of the pro-fibrotic gene *PDGFB* and the extracellular matrix genes: *COL1A1* and *COL3A1* (*Figure 4C*) relative to their WT counterparts. This was accompanied by increased secretion of the pro-fibrotic cytokine TGF-β1, and the extracellular matrix proteins fibronectin-1 and pro-collagen 1 (*Figure 4*). Overexpression of *PDGFA* and *PDGFB* in cardiomyocytes can induce cardiac fibrosis and structural remodelling through paracrine activation of cardiac fibroblasts.^84^ Increased secretion of TGF-β1 observed in co-culture is likely due to pathological interactions between atrial hiPSC-CMs and hiPSC-CFs, with PDGFB acting as a key mediator in the activation of cardiac fibroblasts and the increased propensity for atrial fibrosis.^84,85^

In addition to the electrical remodelling observed, disruption to signal propagation caused by cardiac fibrosis is a common feature of AF^86^ and our data suggests that this is likely to be a prominent mechanism underlying the AF associated TTNtv investigated.

## 5. Conclusions

In conclusion, our study demonstrates that the TTNtv *TTN* c.59926+1 G>A variant induces significant pro-fibrotic and electrical changes in atrial hiPSC-CMs, contributing to an increased propensity for atrial fibrosis and arrhythmia. We have employed a multidisciplinary approach, integrating *in vitro* and *in silico* models to examine the effects of a TTNtv. Mechanisms identified in 2D iPSC-CM cultures were further validated in EHTs and 3D computational simulations of the human atria. The variant causes dysregulation of genes critical for sarcomere function, resulting in shortened sarcomeres, diminished contractile function and heightened cardiac stress. Electrical remodelling, marked by altered ion channel expression and sodium channel kinetics, as well as increased cholinergic sensitivity, fosters a pro-arrhythmic substrate conducive to atrial fibrillation. Additionally, the observed up-regulation of pro-fibrotic growth factors and extracellular matrix proteins highlights the variant's role in promoting cardiac fibrosis. These findings not only enhance our understanding of the pathophysiological mechanisms underlying TTNtv-associated atrial fibrillation but also suggest potential therapeutic targets for mitigating fibrosis and its arrhythmogenic effects. Future research should focus on validating these targets and exploring their clinical applicability to improve outcomes for patients with TTNtv-related heart disease.

## Supplementary Material

cvag112_Supplementary_Data

## Data Availability

All data associated with this study are present in the paper or in the Supplementary Materials. Bulk RNA sequencing datasets used in this study can be requested from the corresponding author.
