# Supplementary material for: A titin truncating variant linked to atrial fibrillation increases atrial profibrotic signalling and cholinergic sensitivity
Source: Cardiovasc Res. 2026 May 27;122(9):1206–23. doi: 10.1093/cvr/cvag112 (PMC13307571; doi:10.1093/cvr/cvag112)
Supplement: cvag112_Supplementary_Data [file cvag112_supplementary_data.zip › Supplementary Tables and Figures CVR-2024-2019 26FEB2026.docx]

A titin truncating variant linked to atrial fibrillation increases atrial profibrotic signalling and cholinergic sensitivity

Max J Cumberland^1,4*^, Albert Dasí^2^, Naeramit Sontayananon^3^, Alan Marcus^4^, Alex Qin^5^,

Leto Riebel^2^, Jonas Euchner^1,6^, Amar J Azad^1^, Caitlin Hall^1^, Christopher O’Shea^1,15^, James GW Smith^7^, Charikleia Papadopoulou^8,9^, Eric A Miska^8^, Davor Pavlovic^1^, Ellis Patrick^5^, James Chong^4^, Paulus Kirchhof^1,10,11^, Chris Denning^13^, Benjamin Davies^14^, Blanca Rodriguez^2^, Andrew P Holmes^1,12^, Katja Gehmlich^1,3*^

## **Supplementary Tables and Figures**

**Table S1: Taqman Probes**

| Target Gene | Assay ID |
| --- | --- |
| *NPPA* | Hs00383230_g1 (FAM) |
| *NPPB* | Hs00173590_m1 (FAM |
| *ACTN2* | Hs00153809_m1 (FAM) |
| *MYH7* | Hs01110632_m1 (FAM) |
| *AGT* | Hs01586213_m1 (FAM) |
| *ACE2* | Hs01085333_m1 (FAM) |
| *HSPB7* | Hs00205296_m1 (FAM) |
| *COL1A1* | Hs00164004_m1 (FAM) |
| *MYH6* | Hs01101425_m1 (FAM) |
| *COL3A1* | Hs00943786_g1 (FAM) |
| *MYL2* | Hs00166405_m1 (FAM) |
| *MYL7* | Hs01085598_g1 (FAM) |
| *GAPDH* | Endogenous Control (VIC, 4326317E) |

**Table S2: Primers**

| Target Gene | Primer Sequences |
| --- | --- |
| *TTN* (Exon 6-7) | F: GTCCCCATCGCCCATAAGAC  R: GAGGCACTTCAGGACCTGTG |
| *TTN* (Exon 11-12) | F: CCCGGGAGCTCAAGAAGAAA  R: TGACTTTGGGTGTGGCAACT |
| *TTN* (Exon 49-50) | F: TGAGGCCTTGAATGACAGCG  R: GATCACTGGGGCAGCTCTTT |
| *TTN* (Exon 296-297) | F: TGCTACTGTCCAGGGTCTCA  R: CTTCCAGGTCTTCAGGACGC |
| *TTN* (Exon 303-304) (Non qPCR) | F: AACCTGGGCCACCTAGAGAT  R: GGAGACCAAGACCCACAATG |
| *TTN* (Exon 317-318) | F: ACCCGCTTTGAGGTTACTGG  R:CCGCATCAAGTTCTCCCTCA |
| *TTN* (Exon 360-361) | F: GGATCTGTGTCCTCTAGCTGC  R: GAAGCTTCCTCTTTGAGGGCT |
| *GAPDH* (Non qPCR) | F: TGCMTCCTGCACCACCAACT  R: YGCCTGCTTCACCACCTTC |
| *EDN1* | F: CTACTTCTGCCACCTGGACATC R: TCACGGTCTGTTGCCTTTGTGG |
| *CTGF* | F: CTTGCGAAGCTGACCTGGAAGA R: CCGTCGGTACATACTCCACAGA |
| *PDGFA* | F: TAGGGAGTGAGGATTCTTTG  R: CGAGGAATCTCGTAAATGAC |
| *PDGFB* | F: GGGCAGGGTTATTTAATATGG  R: AATCAGGCATCGAGACAG |
| *TGF-β1* | F: AACCCACAACGAAATCTATG  R: CTTTTAACTTGAGCCTCAGC |
| *KCNJ3* | F: GAAACAACTGGGATGACTTG  R: CCCTCTTCTAAGGAAATTACAG |
| *KCNK1* | F: ACTTCACCTCCGCGCTCTTCTT  R: AACAGGAGGGTGAAGGGAATGC |
| *KCND2* | F: GCTACAGTTATGTTCTACGC  R: ATACCCTAGTGTTGTCATGG |
| *GAPDH* (qPCR) | F: TCGGAGTCAACGGATTTG R: CAACAATATCCAACTTTACCAGAG |
| *SCN5A* | F – TCT TCA CAG GCG AGT GTA TTG  R – CAC GAT GAG GAA GGA GAT GAT G |
| *SCN1A* | F – GGA CTG TAT GGA GGT TGC TGG T  R – GCA AGG TTG TCT GCA CTA AAT GAG G |
| *SCN9A* | F – GTG GAA GGA TTG TCA GTT CTG CG  R – GCC AAC ACT AAG GTG AGG TTA CC |
| *KCNJ5* | F – GGA CAC CCC AGA AGT TAG CA  R – CCA ATC TCC ATG TCC TGG TT |
| *KCNA4* | F – GTC CGT CCT GGT CAT CTT AAT C  R – CAG CGA ACC ACA AAC TCA AAG |
| *KCNA5* | F – CGA GGA TGA GGG CTT CAT TA  R – CTG AAC TCA GGC AGG GTC TC |
| *KCNE1* | F – GCA TCA GAT GGA TTT TGA AC  R – CAG GCA CAC CTC TAA ATA TG |
| *KCNJ2* | F – GTC TTG GGA ATT CTG GTT TG  R – GAA CAT GTC CTG TTG CTG |
| *KCNJ8* | F – TCT TTA CCA TGT CCT TCC TC R – AGA CCT GAC ATT AGT CAC AC |
| *CACNA1C* | F – GGA GAG TTT TCC AAA GAG AG  R – TTT GAG ATC CTC TTC TAG CTG |

**Table S3: ELISA Kits**

| Name, Target | Company, Catalog | Type |
| --- | --- | --- |
| Human TGF-beta 1 ELISA Kit, TGF-β1 | RayBiotech, ELH-TGFb1-1 | Sandwich |
| Human Pro-Collagen I alpha 1 DuoSet ELISA, Pro-Collagen 1 | R&D Systems, DY6220-05 | Sandwich |
| Human Fibronectin ELISA Kit, Fibronectin | RayBiotech, ELH-FN1-1 | Sandwich |

**Table S4: Primary and Secondary Antibodies**

| Target | Company, Catalog | Host | Concentration |
| --- | --- | --- | --- |
| MLC-2A (Primary) | Synpatic Systems (311 011) | Mouse | 1:500 |
| MLC-2V (Primary) | Proteintech, 10906-1-AP | Rabbit | 1:500 |
| Phalloidin (F-Actin) (Conjugated) | ThermoFisher Scientific, A22284 | N/A | 1:500 |
| α-actinin (Primary) | Abcam, ab68167 | Rabbit | 1: 500 |
| Titin (T12) (Z-Disc Binding) (Primary) | Fürst et al., 1988 | Mouse | 2 μg/mL |
| Vimentin (Primary) | Abcam, ab45939 | Rabbit | 1:500 |
| Alexa Fluor™ 488 anti-Mouse IgG (Secondary) | ThermoFisher Scientific, A28175 | Goat | 1:500 |
| Alexa Fluor™ 546 anti-Rabbit IgG (Secondary) | ThermoFisher Scientific, A-11035 | Goat | 1:500 |
| DAPI (4',6-Diamidino-2-Phenylindole, Dihydrochloride) (Nuclear Stain) (Conjugated) | ThermoFisher Scientific, D1306 | N/A | 0.1 µg/mL |

**Table S5: Biophysical Parameters of Inward Na^+^ Current in WT and Het Atrial hiPSC-CMs**

| Biophysical Parameter | WT | Het | Test Used | Significance (Welsh’s T Test) |
| --- | --- | --- | --- | --- |
| Inact V50 (mV) | -81.453438 | -78.723333 | Welsh’s T Test | ns |
| Inact K (mV) | 6.819 | 6.934 | Mann-Whitney T test | ns |
| Act V50 (mV) | -30.982188 | -28.658421 | Mann-Whitney U test | * (0.0194) |
| Act K (mV) | 5.73175 | 6.23226316 | Welsh’s T Test | ns |
| Recovery P50 (ms) | 28.3827586 | 20.4978889 | Mann-Whitney U test | ** (0.0037) |


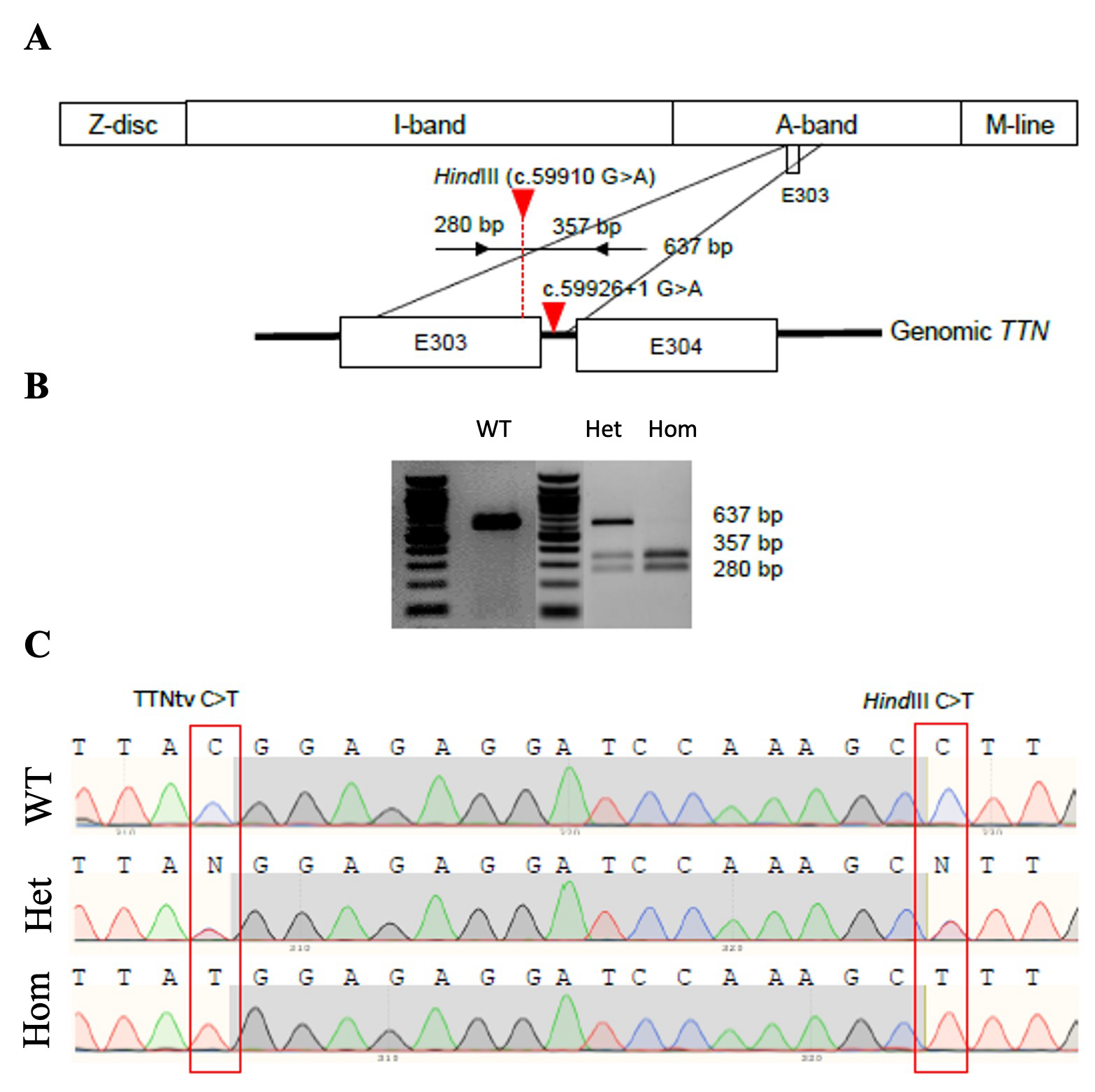


**Figure S1: CRISPR/Cas9-assisted Generation of TTNtv iPSC lines**

(**A**) The A-band TTNtv (TTN c.59926+1 G>A, located within the intron immediately downstream of exon 303), and the silent mutation required to incorporate a *Hin*dIII site (TTN c.59910 G>A) were introduced into an iPSC line by CRISPR/Cas9 targeting. (**B**) PCR amplification of the region surrounding the mutation (637 bp) was performed on isolated iPSC colonies. *Hin*dIII enzyme digestion was performed to assess the genotype of the colonies. The wild-type PCR product (WT/WT) cannot be digested by *Hin*dIII (637 bp). Successful genome editing resulted in the incorporation of the *Hin*dIII site and cleavage of the PCR amplicon (280 and 357 bp). The assay allowed identification of both heterozygous (WT/tv; incomplete digestion) and homozygous clones (tv/tv; complete digestion). (**C**) Sanger DNA sequencing confirmed the genomic changes. The chromatogram presented was derived from the anti-sense strand in the 5’ to 3’ direction as read from left to the right.


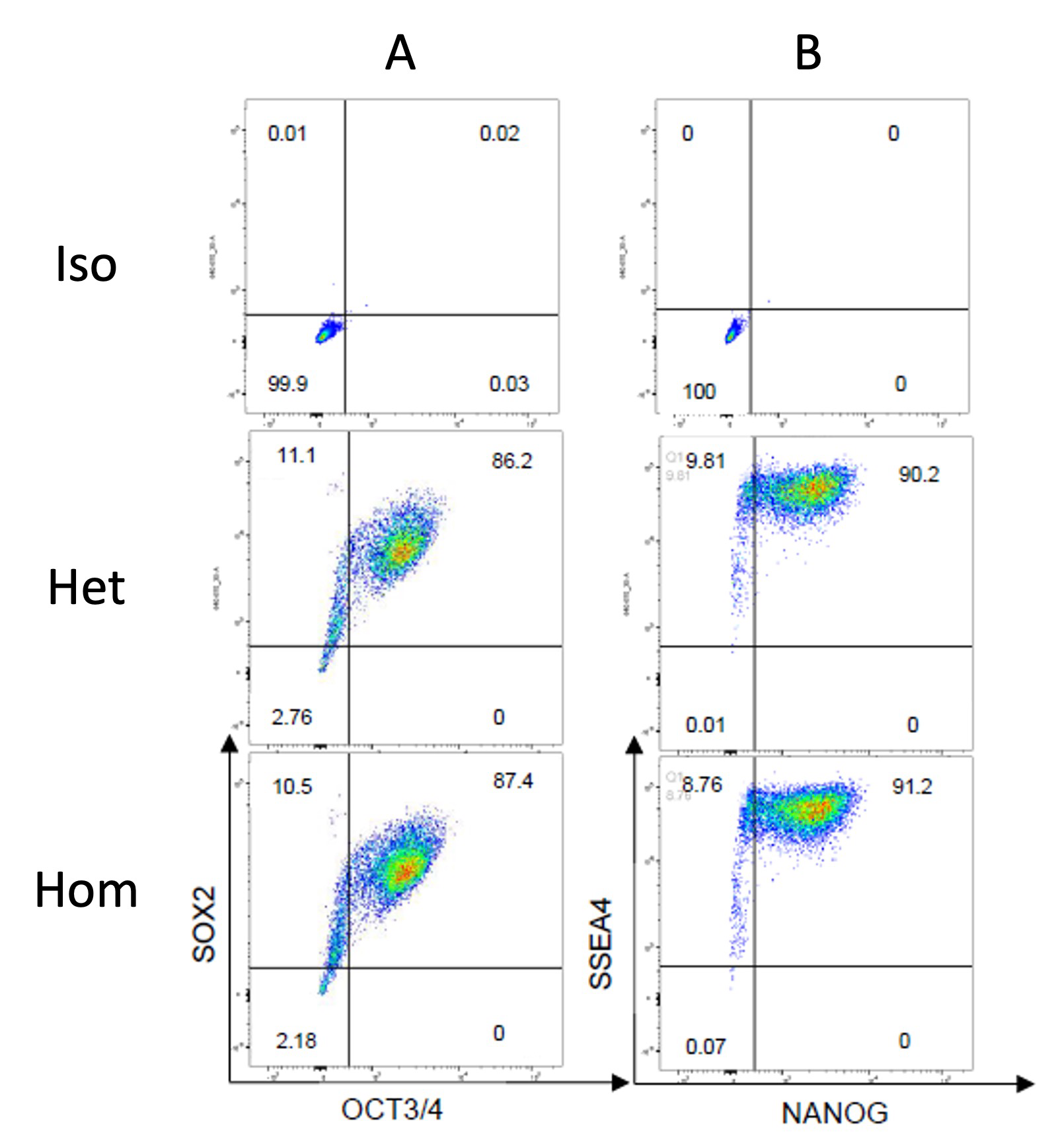


**Figure S2: Analysis of Protein Expression for Pluripotency Markers**
Flow cytometry assays showing expression of pluripotency markers SOX2 and OCT3/4 (**A**) and SSEA4 and NANOG (**B**) for both of the established **Het** and **Hom** iPSC lines. Isotype control is indicated with “**Iso**”. Het and Hom lines qualified as pluripotent stem cells due to a high percentage of expression of the stem cell markers assessed.


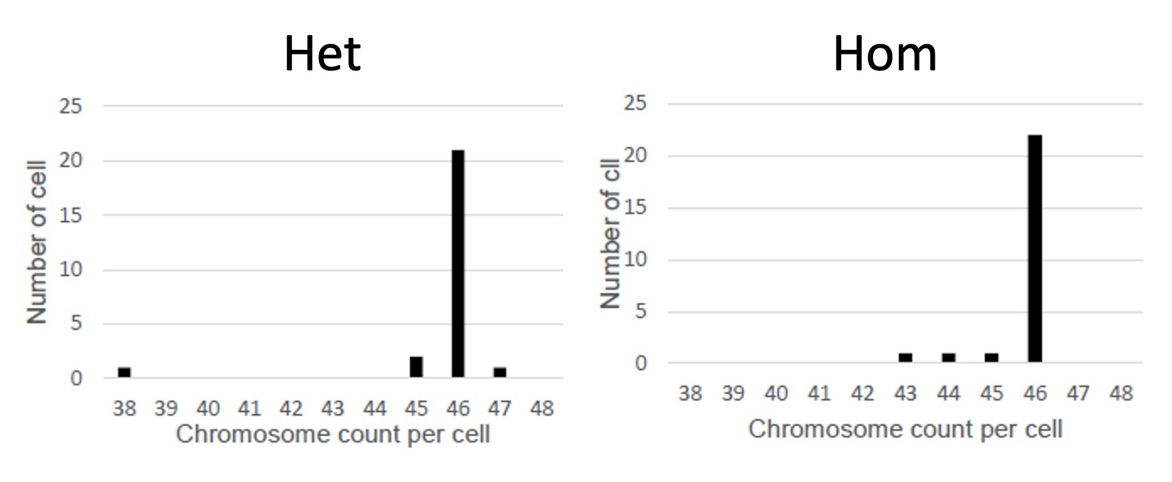


**Figure S3: Karyotype Analysis of TTNtv Het and Hom iPSC lines**
Distribution of chromosome count per cell at the metaphase stage for the established Het and Hom hiPSC lines. Both iPSC lines qualified as karyotypically normal due to the clear modal chromosomal number at 46.


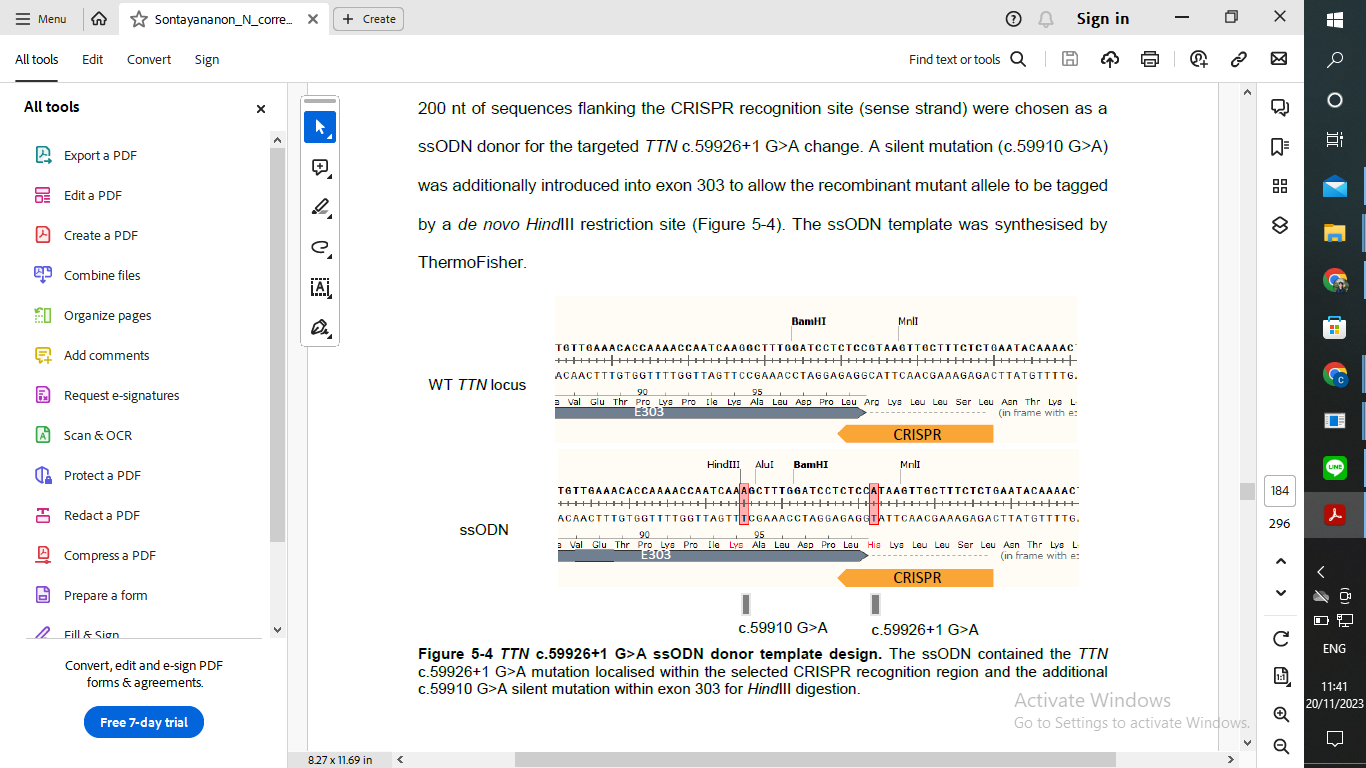


**Figure S4: CRISPR/Cas9 Targeting Strategy to Incorporate TTN c.59926+1 G>A**A guide RNA containing crRNA targeting c.59926+1 (5’-AGAGAAAGCAACUUACGGAG -3’) was complexed with Cas9 protein and electroporated into hiPSCs with a HDR template (showed here as single-stranded oligodeoxynucleotide, ssODN) harbouring both TTNtv (TTN c.59926+1 G>A) and HindIII sites (c.59910 G>A) to generate Het and Hom TTNtv hiPSC lines. The silent mutation corresponding to the HindIII site was introduced for genotypic screening purposes.


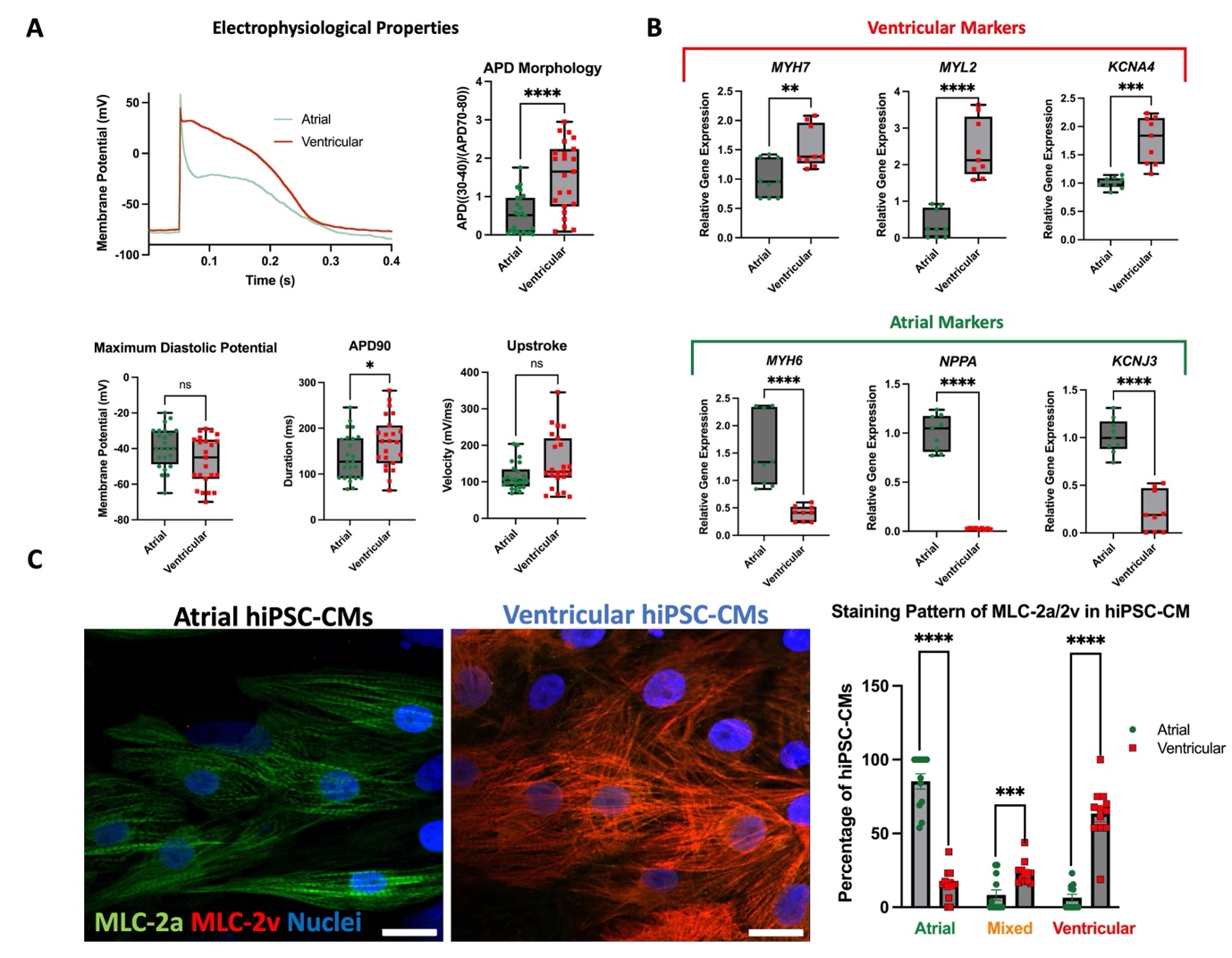


**Figure S5: Characterisation and Validation of Differentiated Atrial hiPSC-CMs**
hiPSCs were differentiated into atrial and ventricular hiPSC-CM and matured until day 30. (**A**) Cells were dissociated, and current clamp was performed, with action potentials stimulated with a 1 nA, 2 ms depolarising current (1 Hz). Upstroke, APD90, Maximum Diastolic Potential and APD Morphology are shown above (Atrial N = 1, n = 23) (Ventricular N = 1, n = 23). A Welch’s t test was used to ascertain significance in the Maximum Diastolic Potential and APD90 (* denotes P < 0.05, **** denotes P < 0.0001). A Mann-Whitney U test was used to ascertain significance in APD Morphology and Upstroke. (**B**) RT-qPCR was performed on the atrial and ventricular specific gene markers MYH7, MYL2, KCNA4, MYH6, NPPA, KCNJ3, MYL7 (N =3, n=9). A Welch’s t test was used to ascertain significance in MYH7, MYL2, KCNA4, MYH6, NPPA, KCNJ3 (* denotes P < 0.05, ** denotes P < 0.01, **** denotes P < 0.0001). A Mann-Whitney U test was used to ascertain significance in MYL7. (**C**) Atrial and Ventricular hiPSC-CMs were fixed and stained for the atrial and ventricular specific isoforms of Myosin Light Chain (MLC-2a/MLC-2v) (Scale Bar = 20 microns). Images were analysed for the percentage of cells positive for each and both markers (N = 1 Differentiation Batch, n = 12 images from 1 coverslip). A Welch’s t test was used to ascertain significance in the number of cells positive for each or both of the markers (*** denotes P < 0.001, **** denotes P < 0.0001).


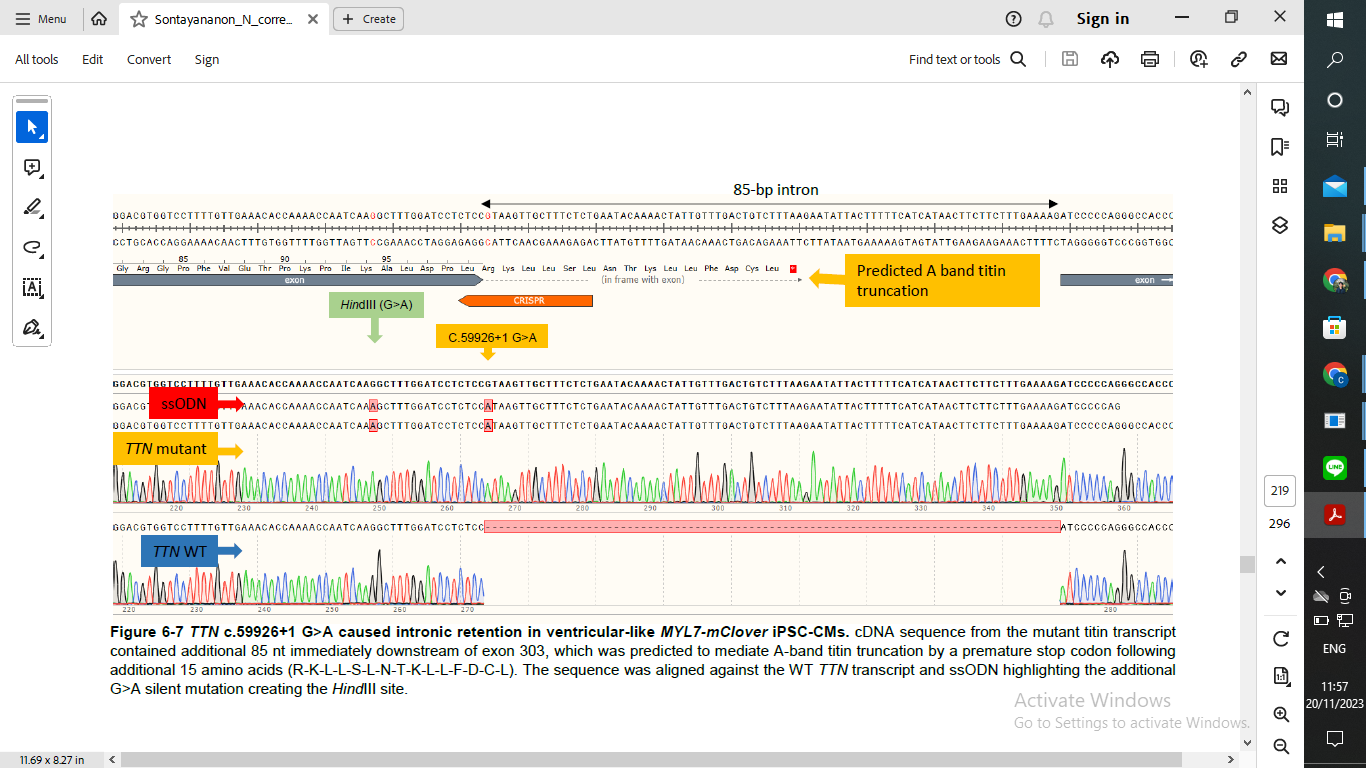


**Figure S6: Retention of Downstream Intron in TTN c.59926+1 G>A**DNA sequencing of the mutant TTN cDNA derived from the transcript carrying TTN c.59926+1 G>A showed additional nucleotides (85 bp) corresponding to the intron immediately downstream of exon 303 which was absent in the WT transcript. Retention of the downstream intron introduced a frameshift and premature stop codon (TTN p.H10911RfsX16).


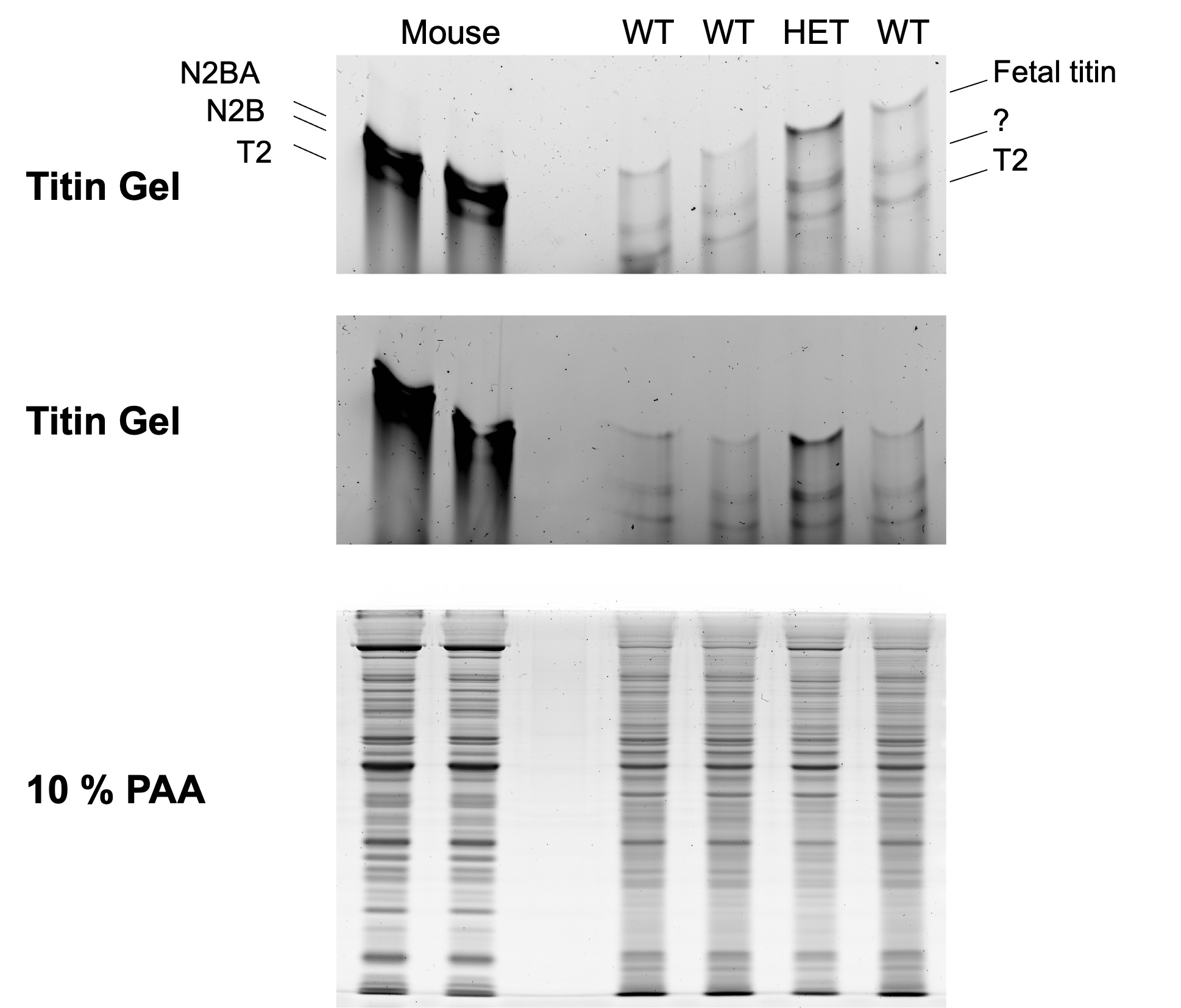


**Figure S7: Titin Gel**Separation of titin bands by agarose-supported poly-acrylamide (PAA) gel electrophoresis, stained with SYPRO-Ruby. Two attempts are shown (top and middle). Mouse ventricular tissue served as positive control, and N2BA, N2B and T2 band are indicated. For atrial hiPSC-CMs, titin runs as a larger foetal isoform in agreement with the foetal like status of hiPSC-CMs. Additional bands are potential splice isoforms (‘?’) and the T2 band. No differences are observed between WT and Het sample. A standard 10 % PAA gel (bottom) was used visualised myofibrillar proteins and serves as loading control.


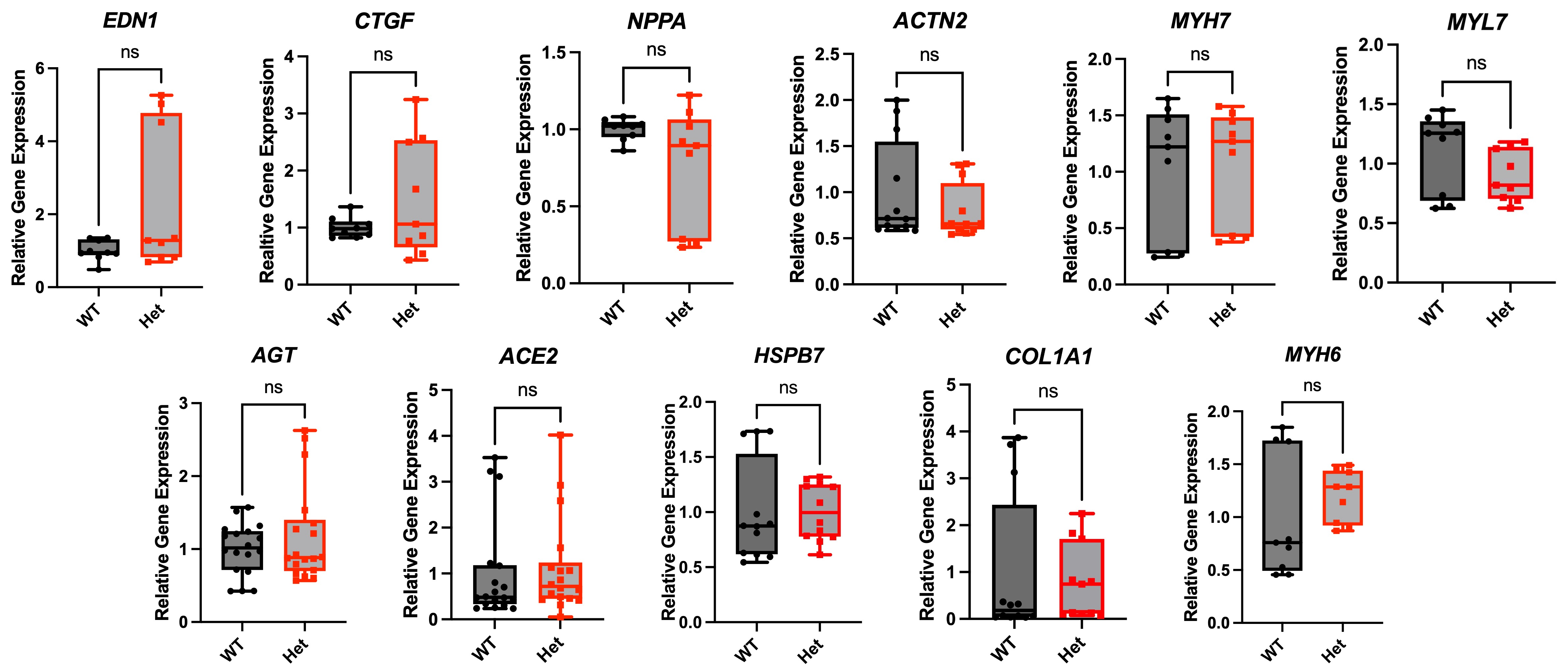


**Figure S8: Cardiac Structural and Stress Markers**
RT-qPCR was performed on the RNA of WT and Het atrial hiPSC-CMs using either primers or TaqMan probes designed to amplify EDN1, CTGF, NPPA, ACTN2, MYH7, AGT, ACE2, HSPB7, COL1A1 and MYH6 (N > 3, n > 9). A Welch’s t-test was used to test significance of ACTN2, MYH7, MYL7, and MYH6 (ns denotes P > 0.05). A Mann-Whitney U-test were used to ascertain significance of EDN1, CTGF, NPPA, AGT, ACE2, HSPB7, and COL1A1 (ns denotes P > 0.05).


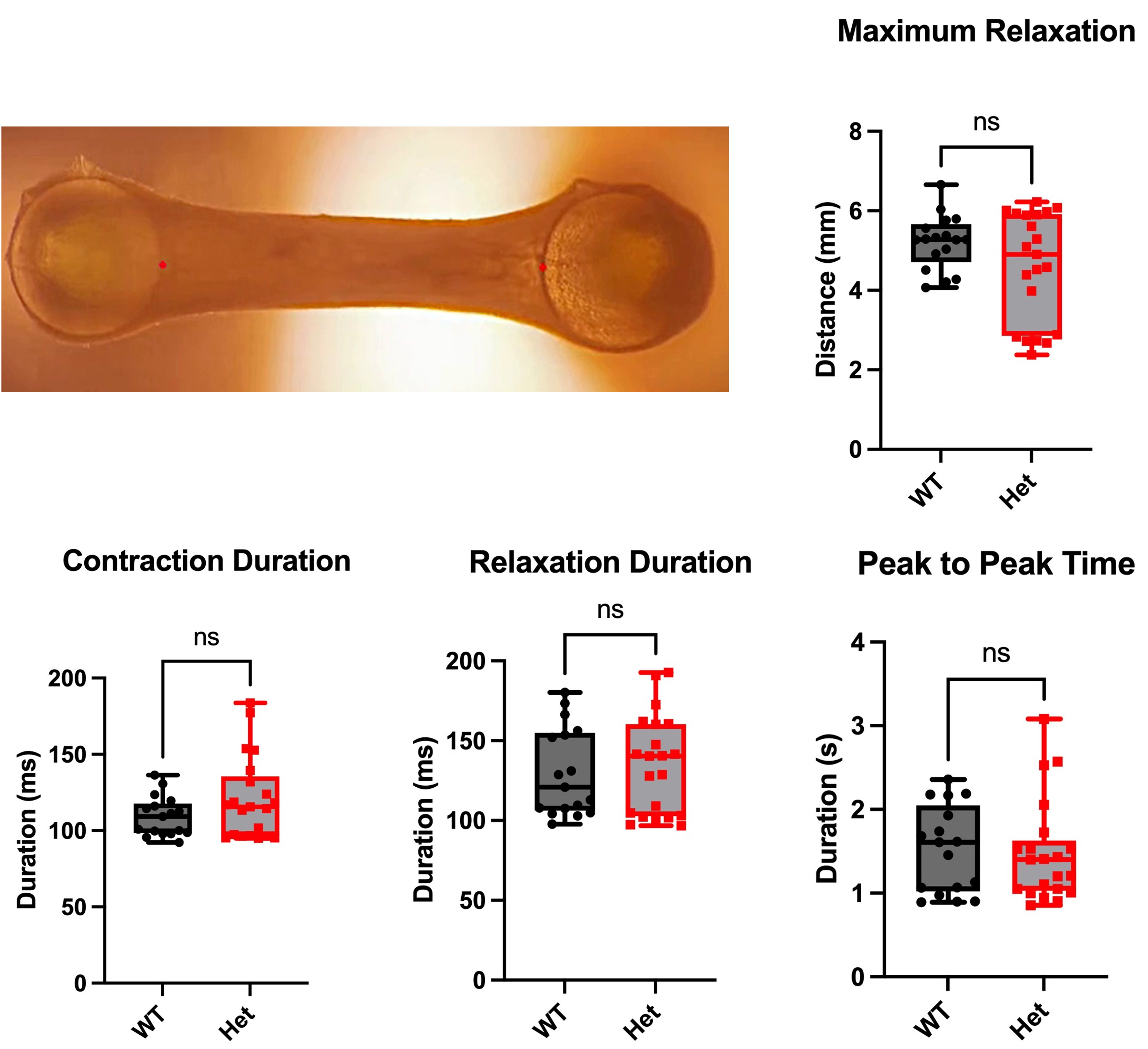


**Figure S9: Contractile Analysis of WT and Het TTNtv Engineered Heart Tissues**
Videos of day 35 EHTs were recorded and the movements of the pillars during EHT beating were tracked with CoTracker3 using a bespoke script (**top left**). Videos were analysed for maximum relaxation (maximum inter-pole distance during relaxation), contraction duration, relaxation duration, and peak-to-peak time in WT (N = 5 EHT batches, n = 17 EHTs) and Het (N = 5, n = 21). Linear mixed and simple models were used to ascertain significance (ns denotes P > 0.05).


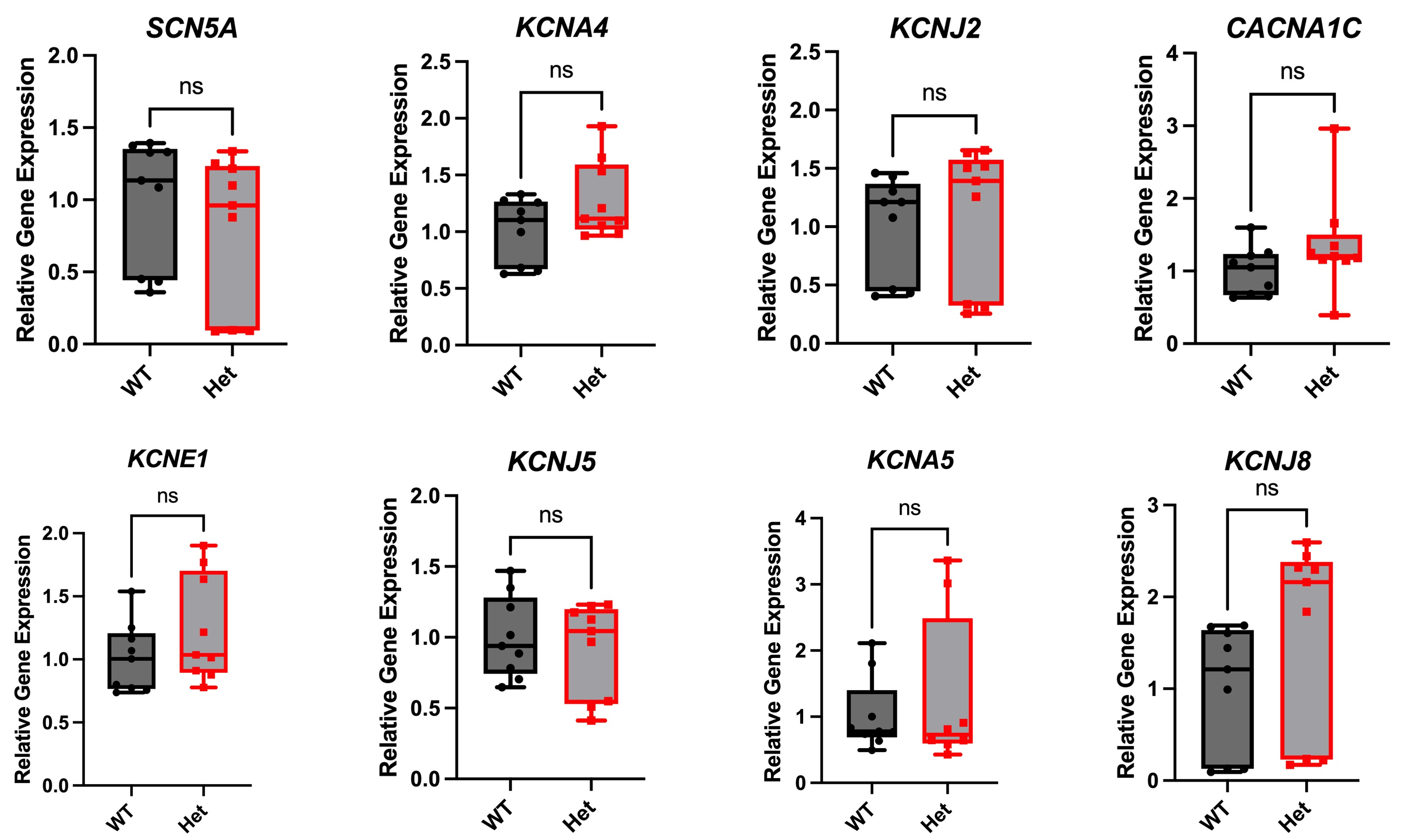


**Figure S10: Gene Expression of Ion Channel Subunit genes in WT and Het Atrial hiPSC-CM**
RT-qPCR was performed on RNA from WT and Het atrial hiPSC-CMs using primers designed to amplify ion channel subunit genes: SCN5A, KCNA4, KCNJ2, CACNA1C, KCNE1, KCNJ5, KCNA5, KCNJ8 (N = 3, n = 9). A Welch’s t test was used to ascertain significance in all genes except for CACNA1C, where a Mann-Whitney U test was used (ns denotes P > 0.05).


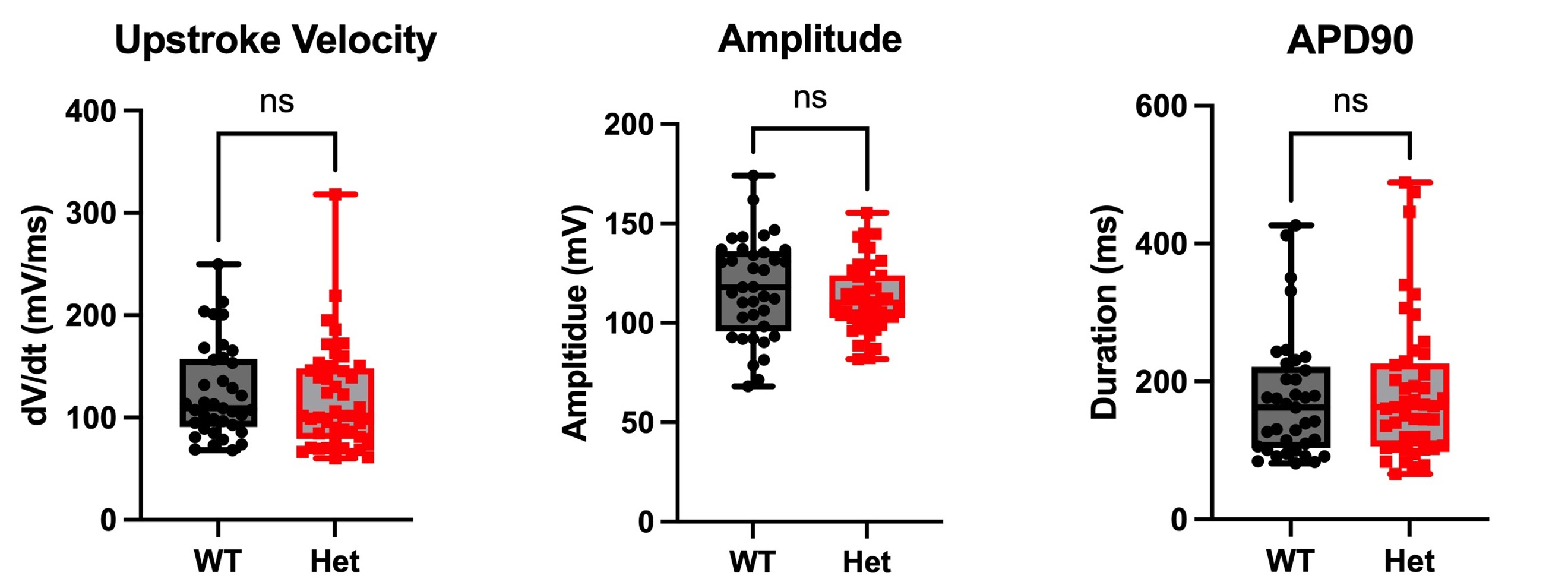


**Figure S11: Stimulated Action Potentials of WT and Het Atrial hiPSC-CMs**
Current clamp was performed on WT and Het atrial hiPSC-CMs, with action potentials stimulated at 1 Hz using a 1 nA (2 ms) depolarising current. Upstroke, Amplitude and Action Potential Duration at 90 % repolarisation (APD90) are shown above. WT, (N = 3, n = 37) and Het (N = 3, n = 45). A Mann-Whitney U-test was used to ascertain significance in the Upstroke Velocity and the APD90, a Welch’s t-test was used to ascertain significance in the Amplitude (ns denotes P > 0.05).

**
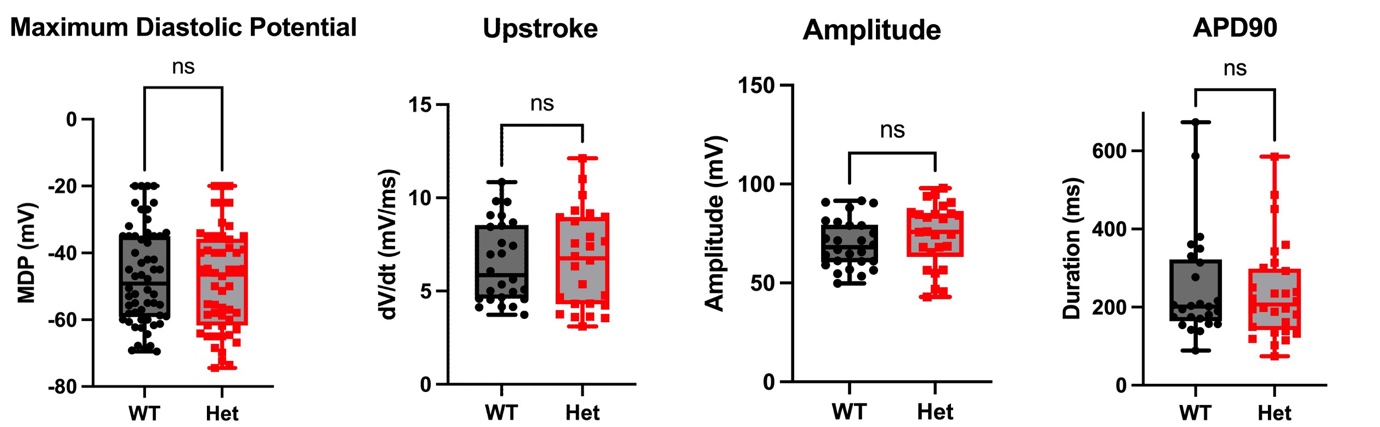
**

**Figure S12: Spontaneous Action Potentials of WT and Het Atrial hiPSC-CMs**
Current clamp was performed on WT and Het hiPSC-CMs. Spontaneous action potentials were recorded over a 60 second period. Maximum Diastolic Potential (MDP), Upstroke, Amplitude and Action Potential Duration at 90 % repolarisation (APD90) are shown above. Maximum Diastolic Potential WT, N = 3, n = 58 and Het, N = 3, n = 54. Upstroke, Amplitude and APD90: WT, N = 3, n = 26 and Het, N = 3, n = 26. A Mann-Whitney U-test was used to ascertain significance for Maximum Diastolic Potential and APD90. Welch’s t-test was used to test significance for Upstroke Velocity and Amplitude (ns denotes P > 0.05).


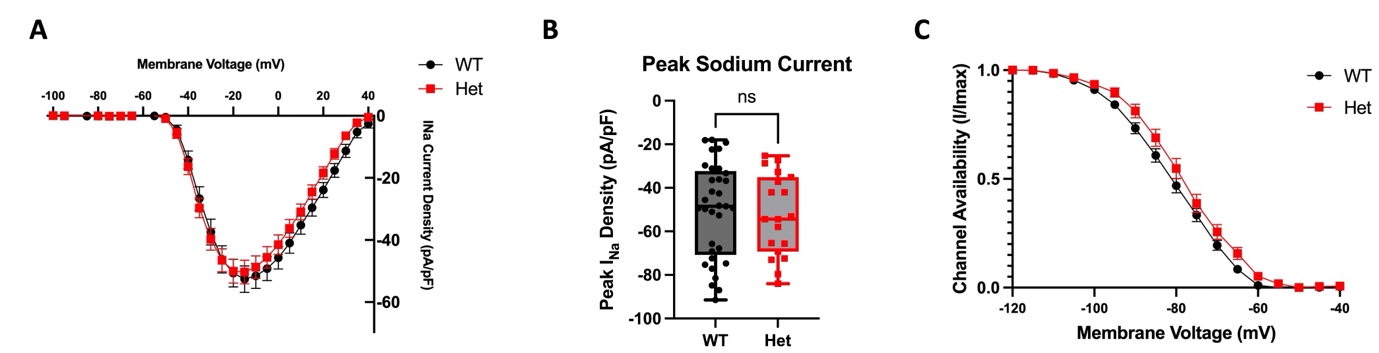


**Figure S13: Inward Na^+^ Current of WT and Het Atrial hiPSC-CMs**
Voltage clamp was performed on day 30 WT and Het atrial hiPSC-CMs to assess inward sodium current amplitude and kinetics. (**A**) Current density – voltage (I-V) graph of Na_v_ channels, (**B**) Peak I_Na+_ Density, (**C**) Voltage dependence of inactivation. WT, N = 3, n = 33 and Het, N = 3, n = 19. A Welch’s t-test was used to test significance (ns denotes P > 0.05).


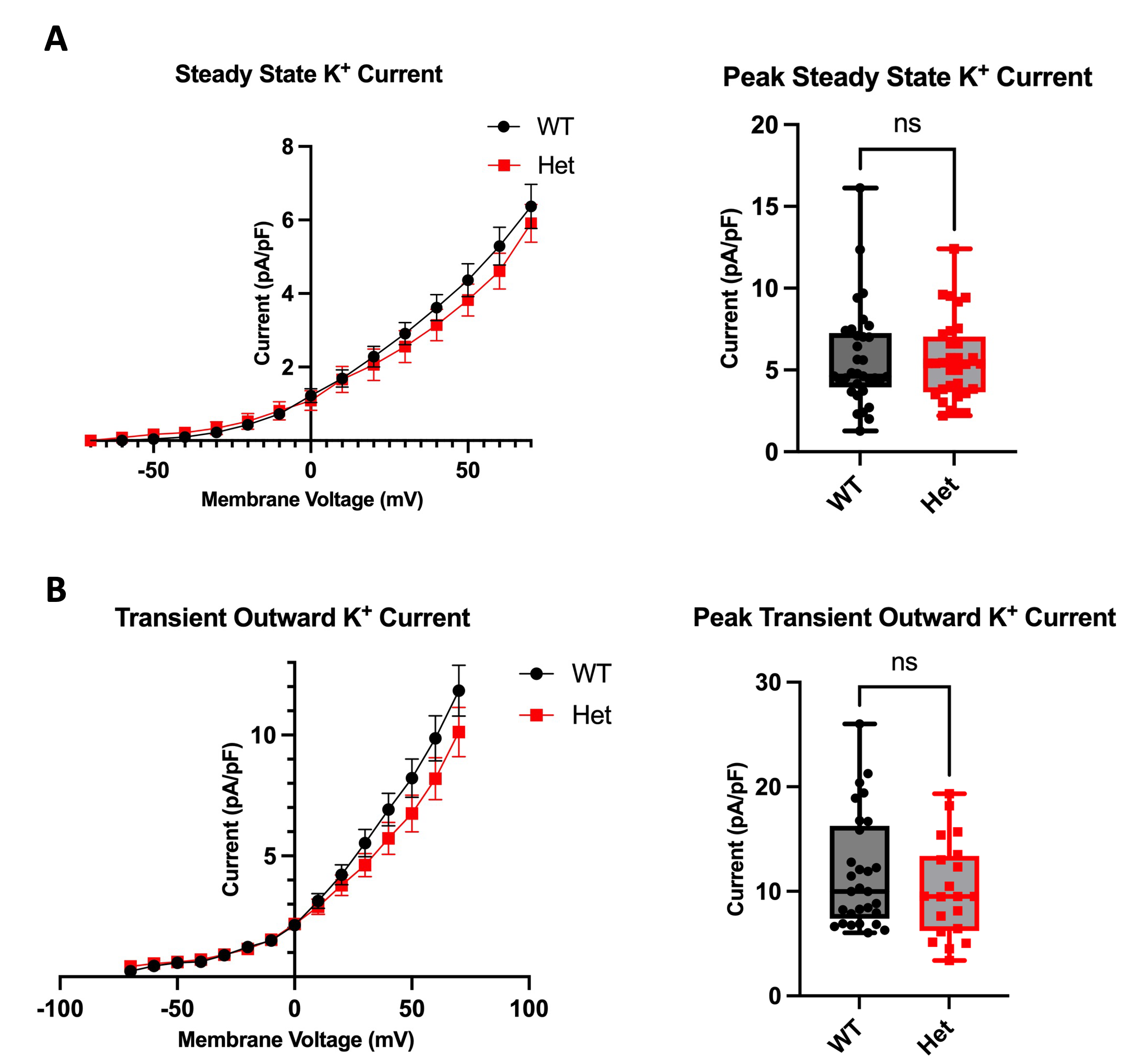


**Figure S14: Outward K^+^ Current of WT and Het Atrial hiPSC-CMs**
Voltage clamp was performed for peak steady state and transient outward K^+^ current. (**A**) Current density – voltage (I-V) graph and peak steady state K^+^ current (WT, N = 3, n = 33) (Het, N = 3, n = 32) (**B**) Current density – voltage (I-V) graph and peak transient outward K^+^ current (WT, N = 3, n = 29) (Het, N = 3, n = 20). A Mann-Whitney U-test was used to ascertain significance of Peak Steady State K^+^ current. A Welch’s t-test was used to test significance in Peak Transient Outward K^+^ current (ns denotes P > 0.05).


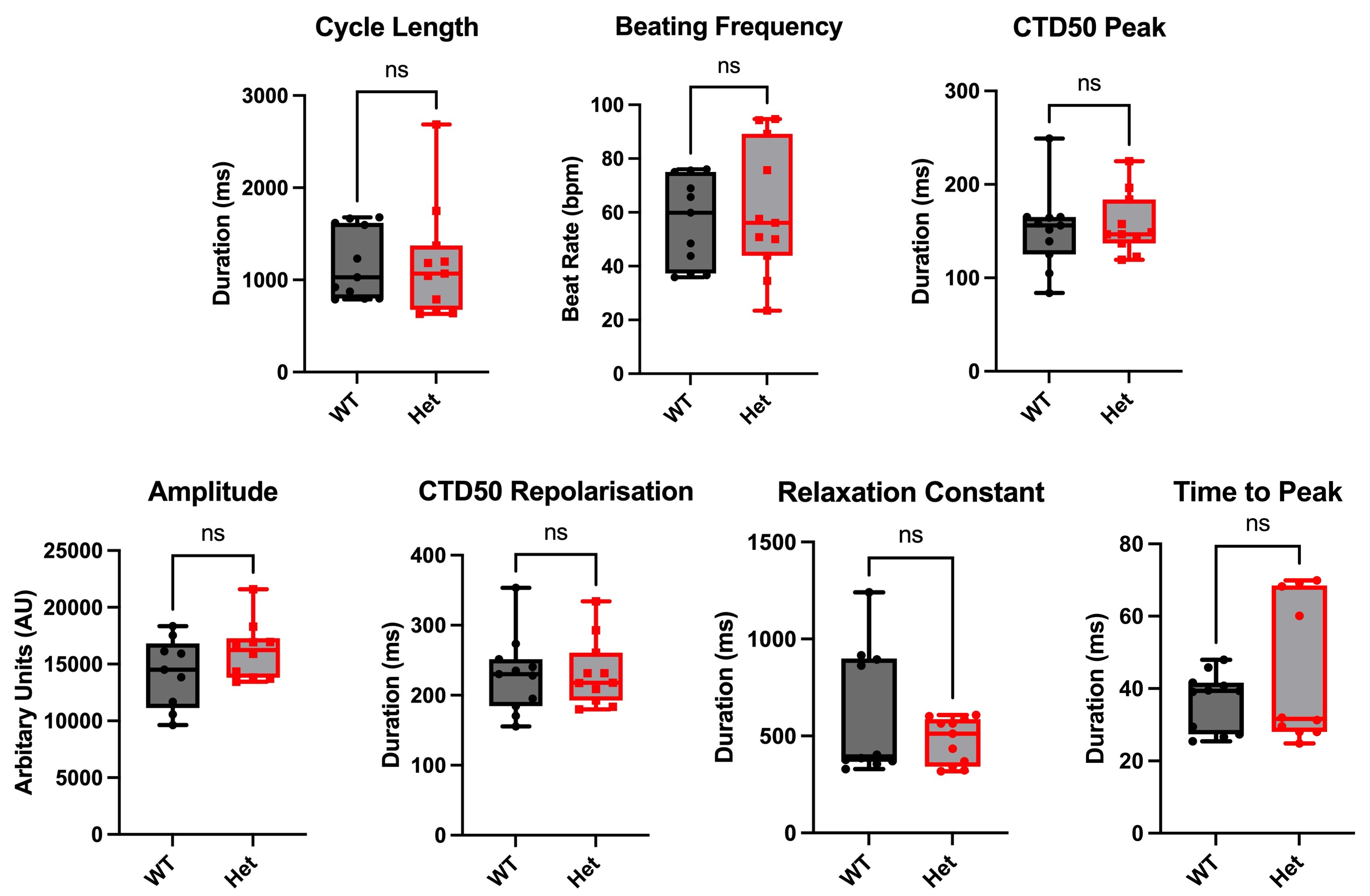


**Figure S15: Optical Mapping of Calcium Transients**Calcium imaging output parameters in WT and Het atrial hiPSC-CMs: Cycle Length, Beating Frequency, CTD50 Peak, Amplitude and CTD50 Repolarisation, Relaxation Constant and Time to Peak (N = 3, n ≥ 9). A Welch’s t-test was used to test significance in all parameters except Cycle Length and Time to Peak where a Mann-Whitney U test was used (ns denotes P > 0.05).


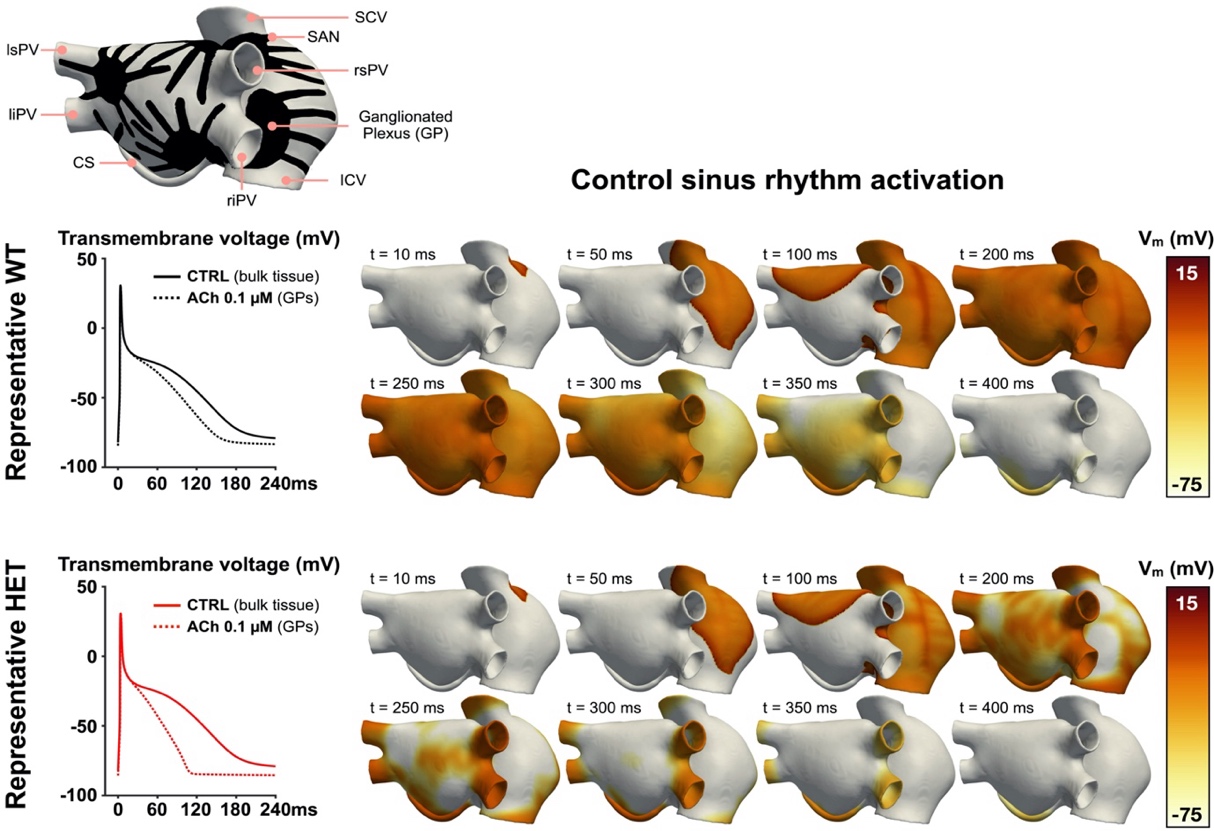


**A**

**B**

**Figure S16: Heterogeneous Repolarisation for TTNtv Het Conditions during Sinus Rhythm
(A)**: Schematic representation of the human whole-atria model used for multi-scale simulations. Abbreviations. SAN: sinoatrial node; SCV-ICV: superior and inferior cava vein; rs-ri-ls-li-PV: right superior, right inferior, left superior and left inferior pulmonary vein; CS: coronary sinus. The release of 0.1 μM acetylcholine only occurs at the ganglionated plexuses (GPs), arranged following an octopus configuration. (**B)**: Atrial cardiomyocyte models used for WT and Het conditions. (left). Consecutive snapshots of the atrial transmembrane voltage (V_m_) during control sinus rhythm activation. The considerably shorter APD in GPs sites for Het conditions creates a heterogeneous repolarisation (right).
