# Supplementary figures and images for: A titin truncating variant linked to atrial fibrillation increases atrial profibrotic signalling and cholinergic sensitivity
Source: Cardiovasc Res. 2026 May 27;122(9):1206–23. doi: 10.1093/cvr/cvag112 (PMC13307571; doi:10.1093/cvr/cvag112)

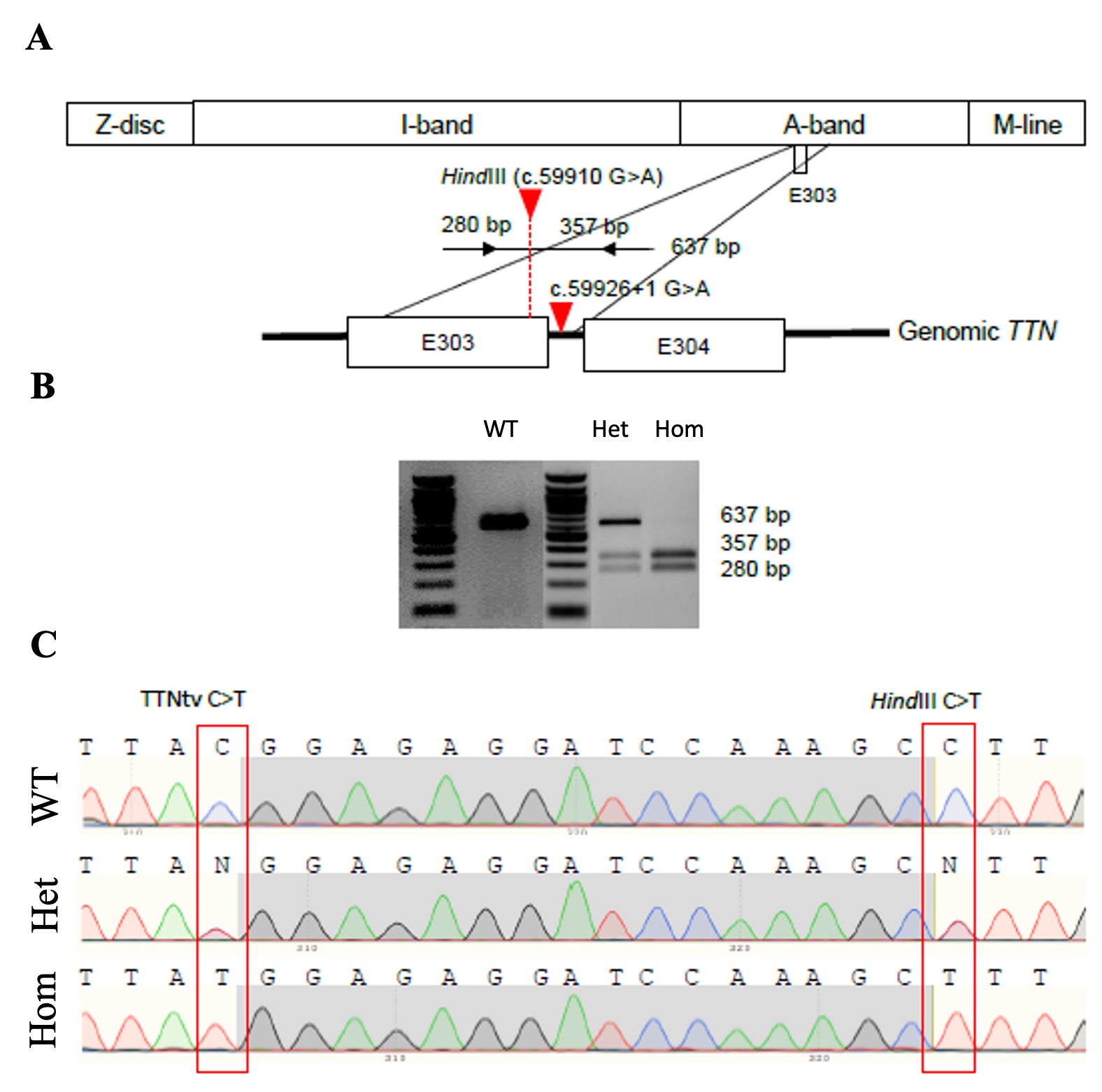

Supplement: cvag112_Supplementary_Data [file cvag112_supplementary_data.zip › Figure S1.jpg]

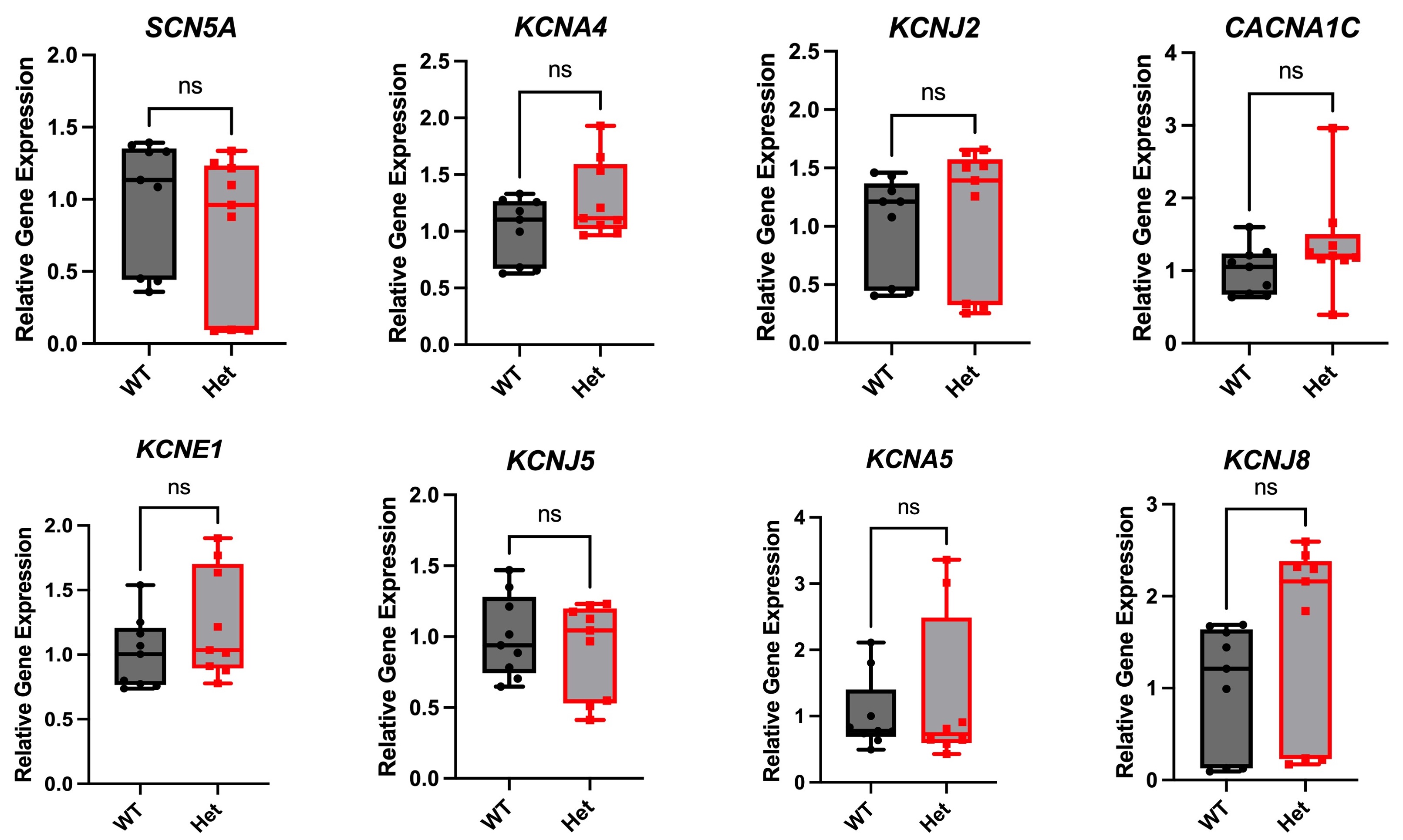

Supplement: cvag112_Supplementary_Data [file cvag112_supplementary_data.zip › Figure S10.jpg]

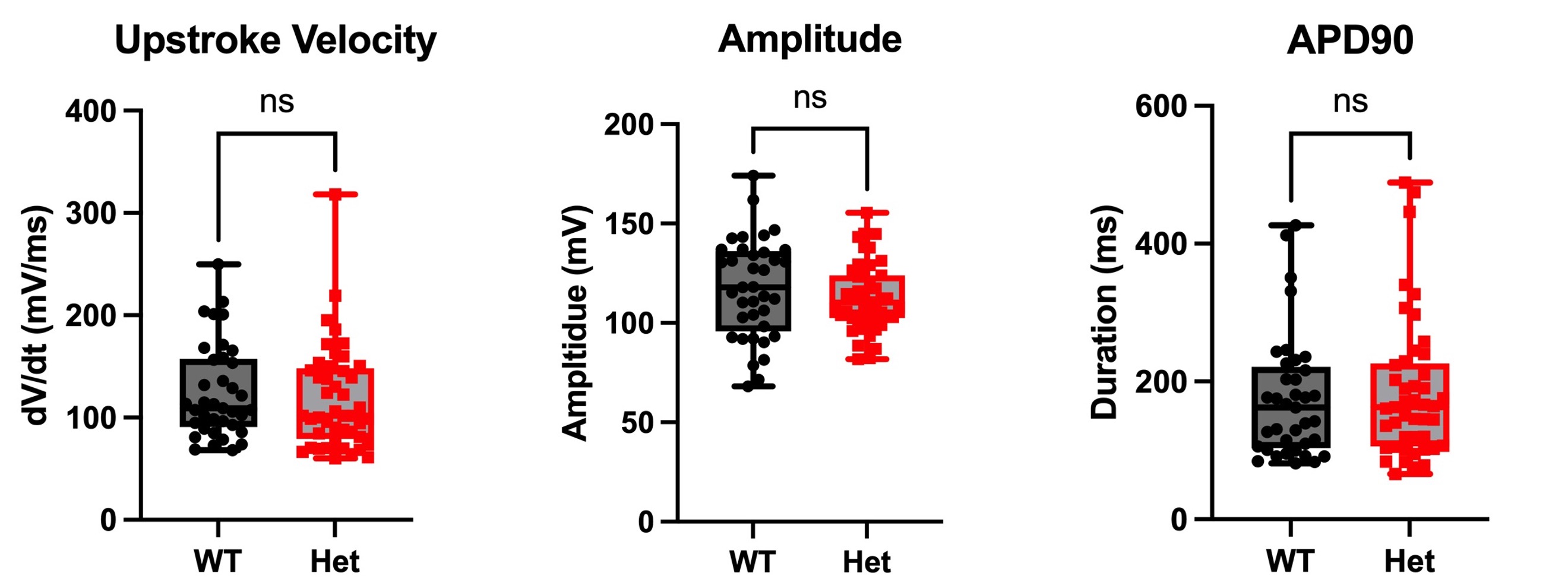

Supplement: cvag112_Supplementary_Data [file cvag112_supplementary_data.zip › Figure S11.jpg]

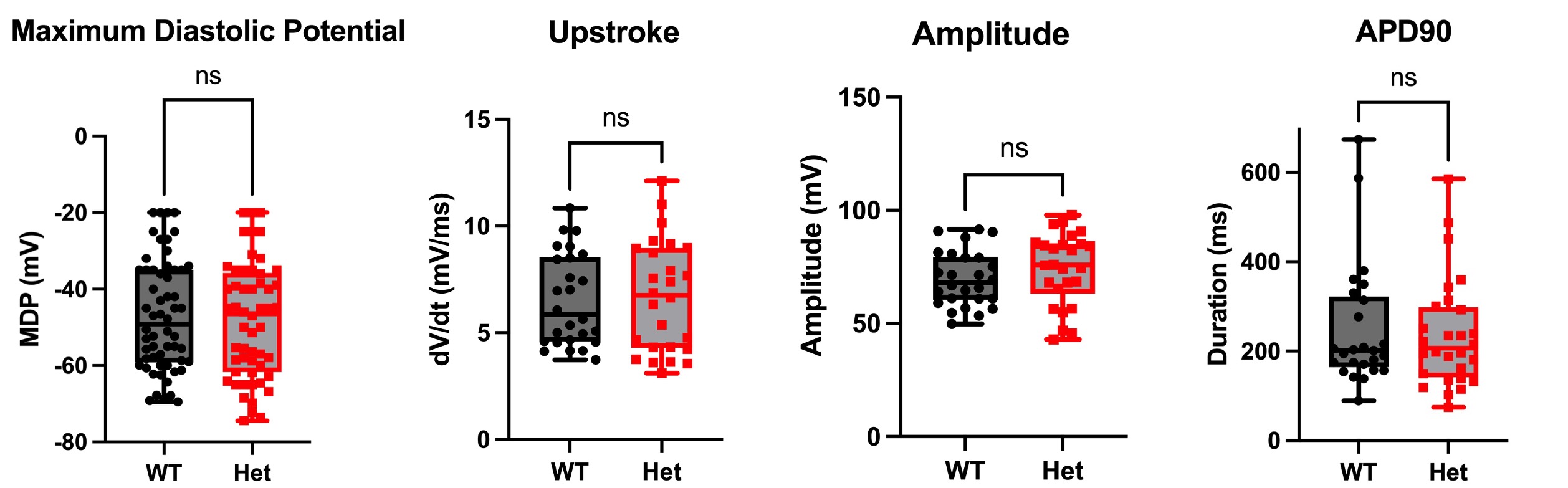

Supplement: cvag112_Supplementary_Data [file cvag112_supplementary_data.zip › Figure S12.jpg]

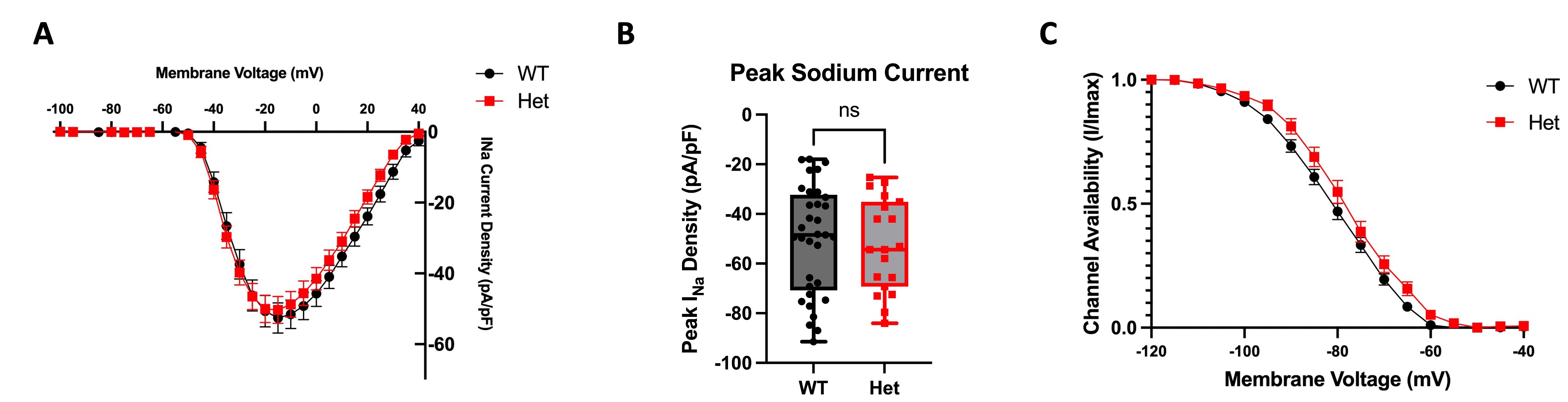

Supplement: cvag112_Supplementary_Data [file cvag112_supplementary_data.zip › Figure S13.jpg]

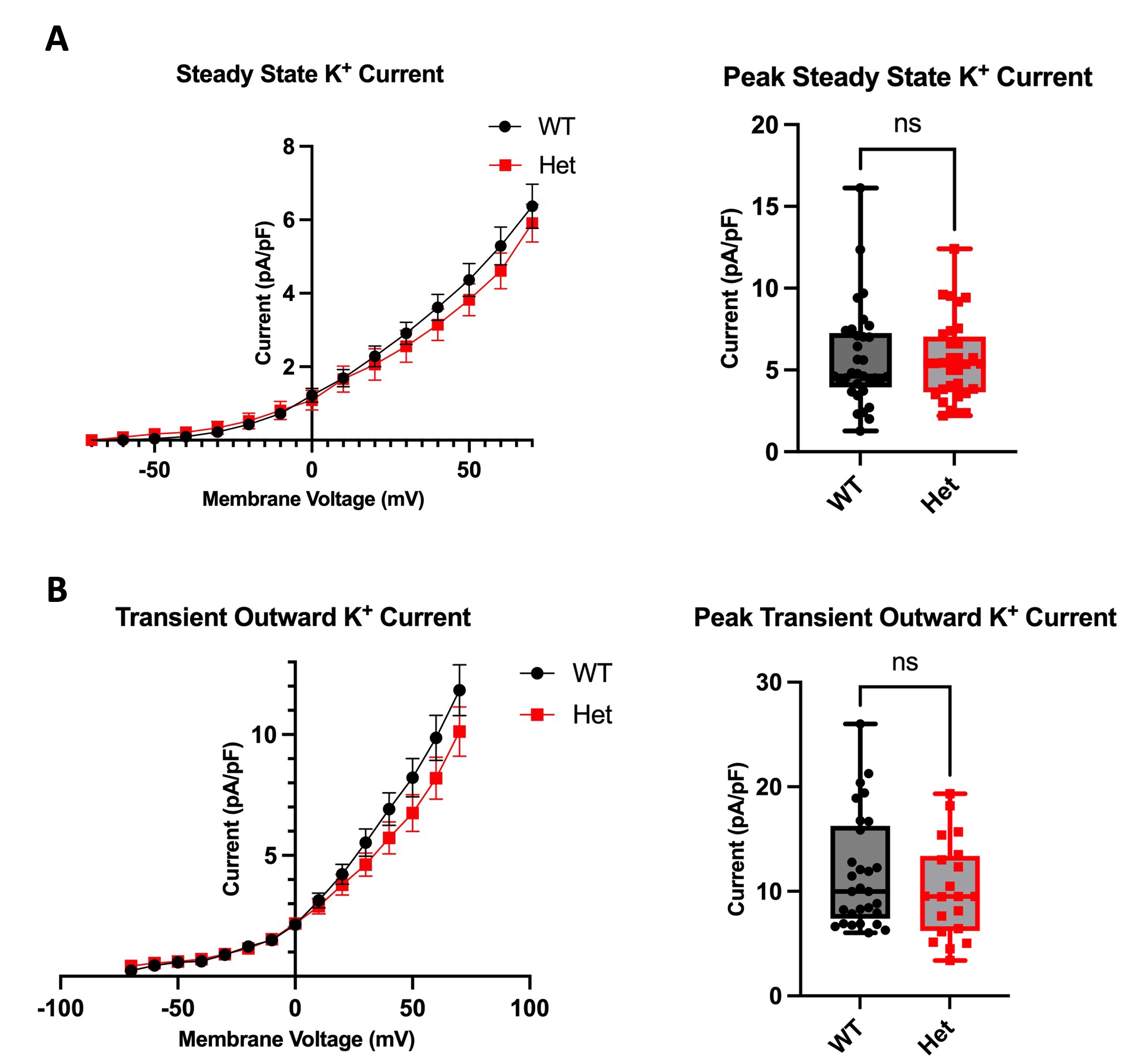

Supplement: cvag112_Supplementary_Data [file cvag112_supplementary_data.zip › Figure S14.jpg]

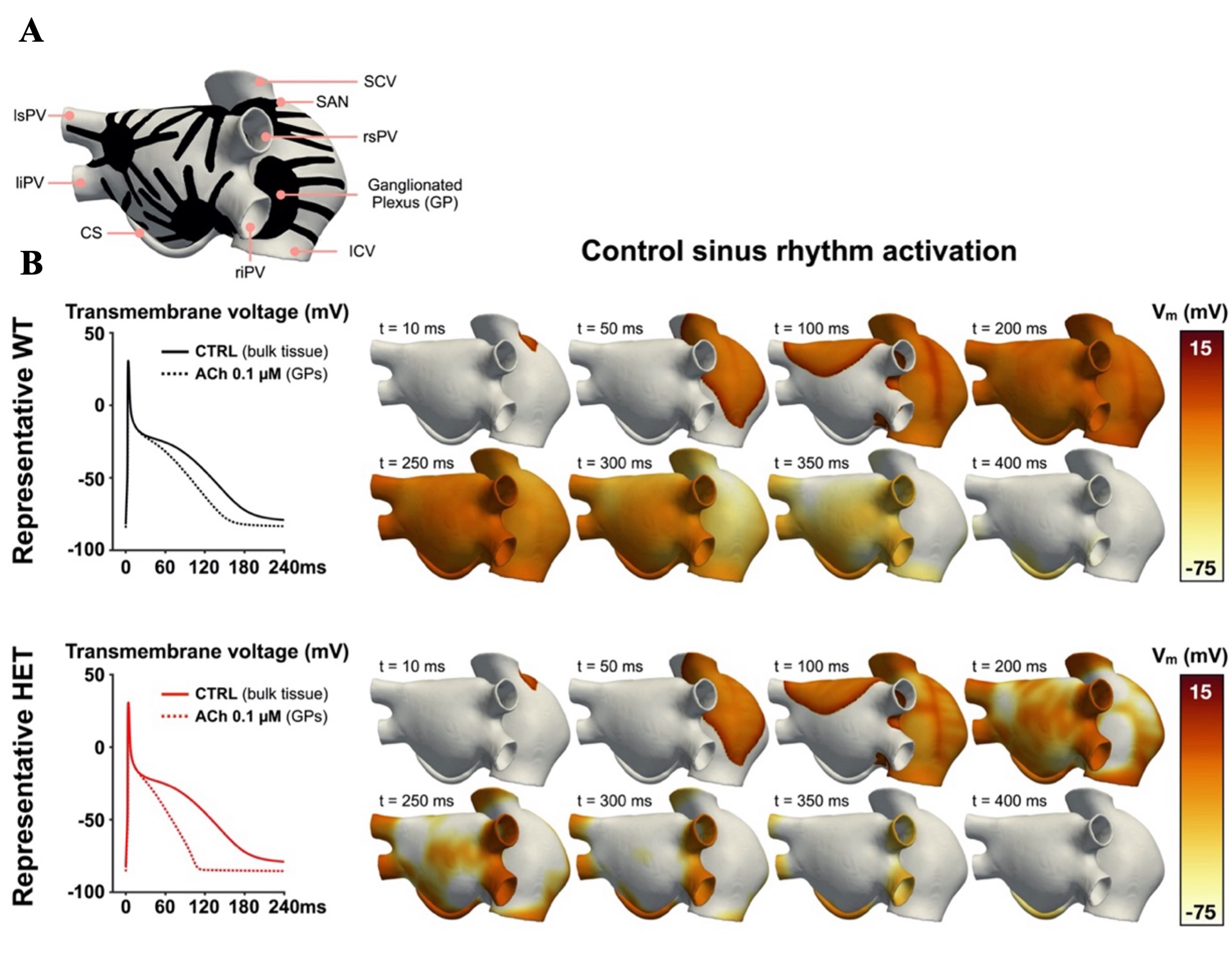

Supplement: cvag112_Supplementary_Data [file cvag112_supplementary_data.zip › Figure S16.jpg]

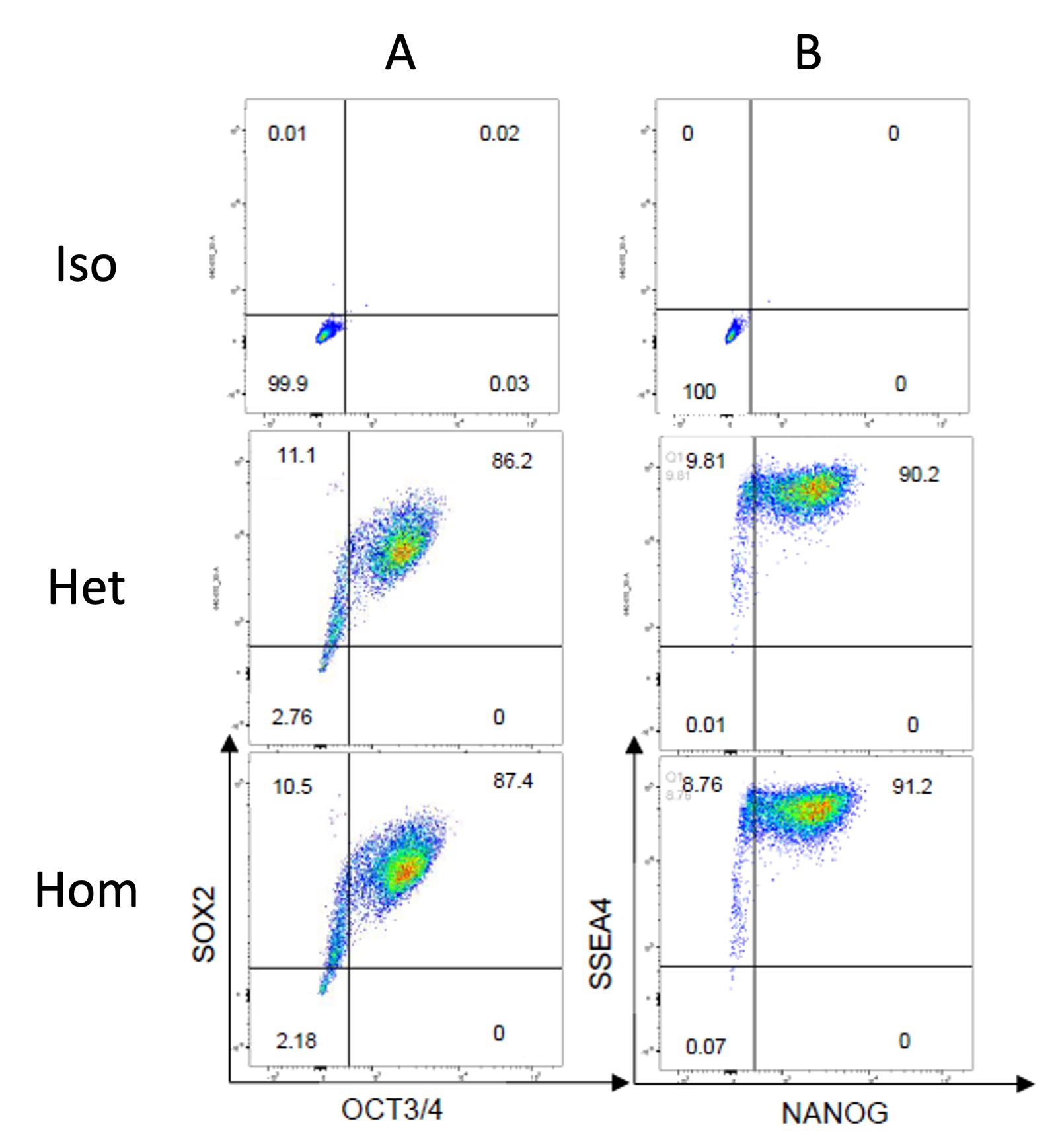

Supplement: cvag112_Supplementary_Data [file cvag112_supplementary_data.zip › Figure S2.jpg]

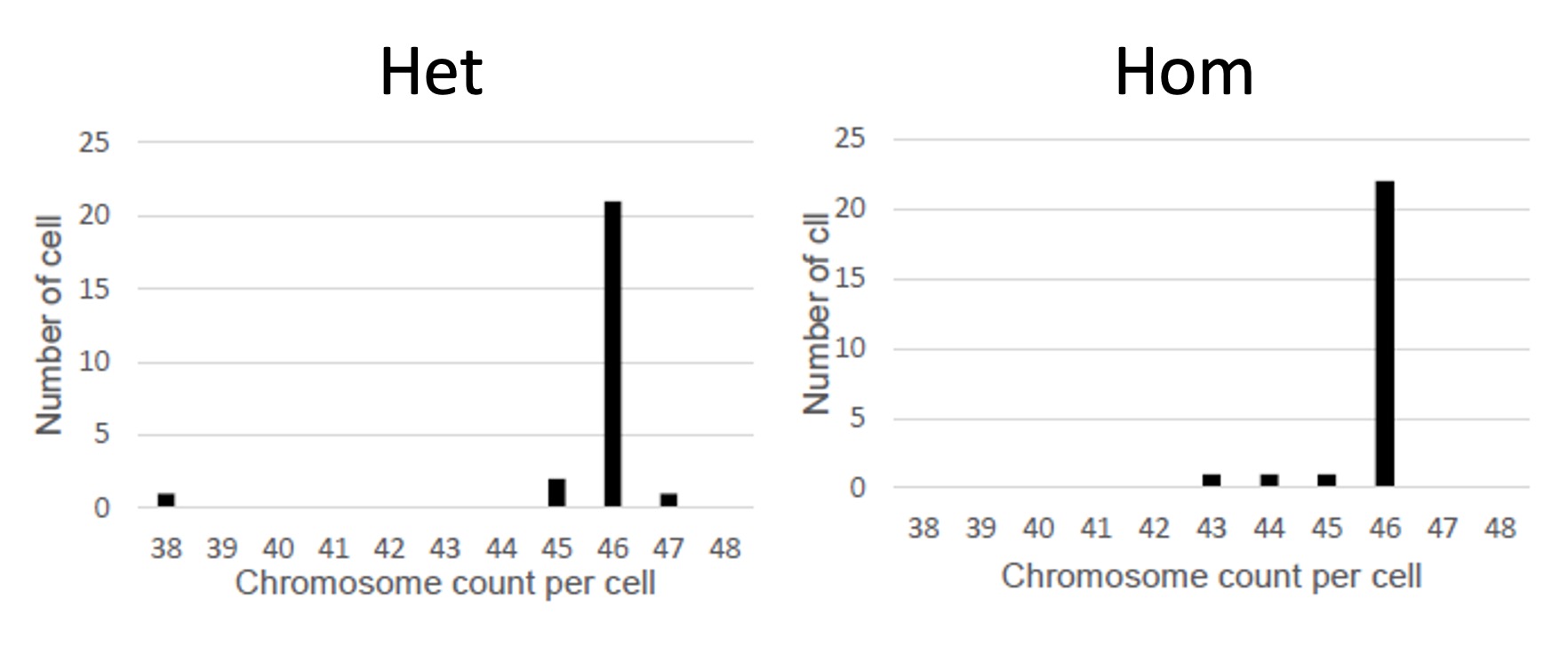

Supplement: cvag112_Supplementary_Data [file cvag112_supplementary_data.zip › Figure S3.jpg]

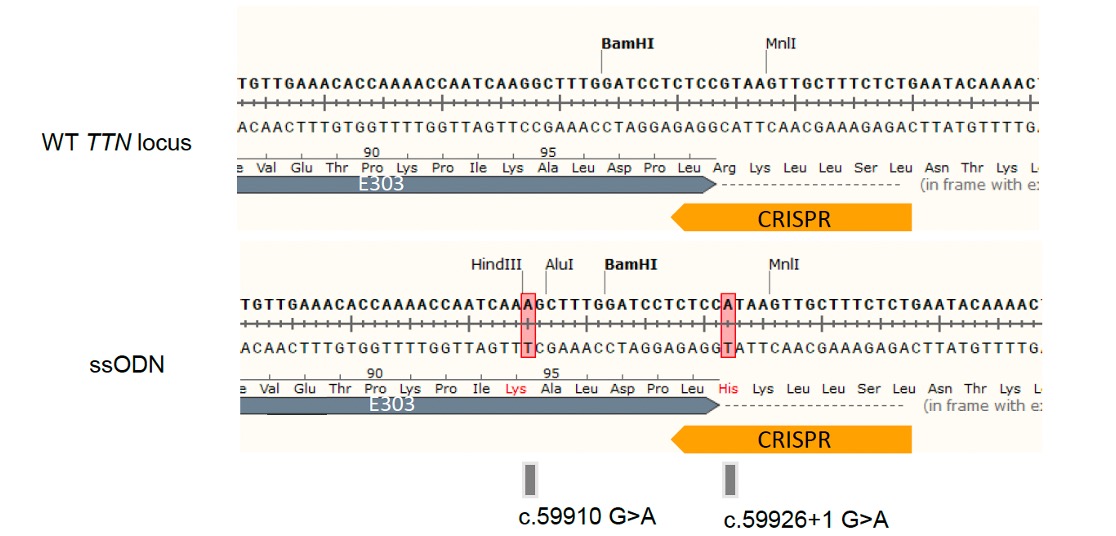

Supplement: cvag112_Supplementary_Data [file cvag112_supplementary_data.zip › Figure S4.jpg]

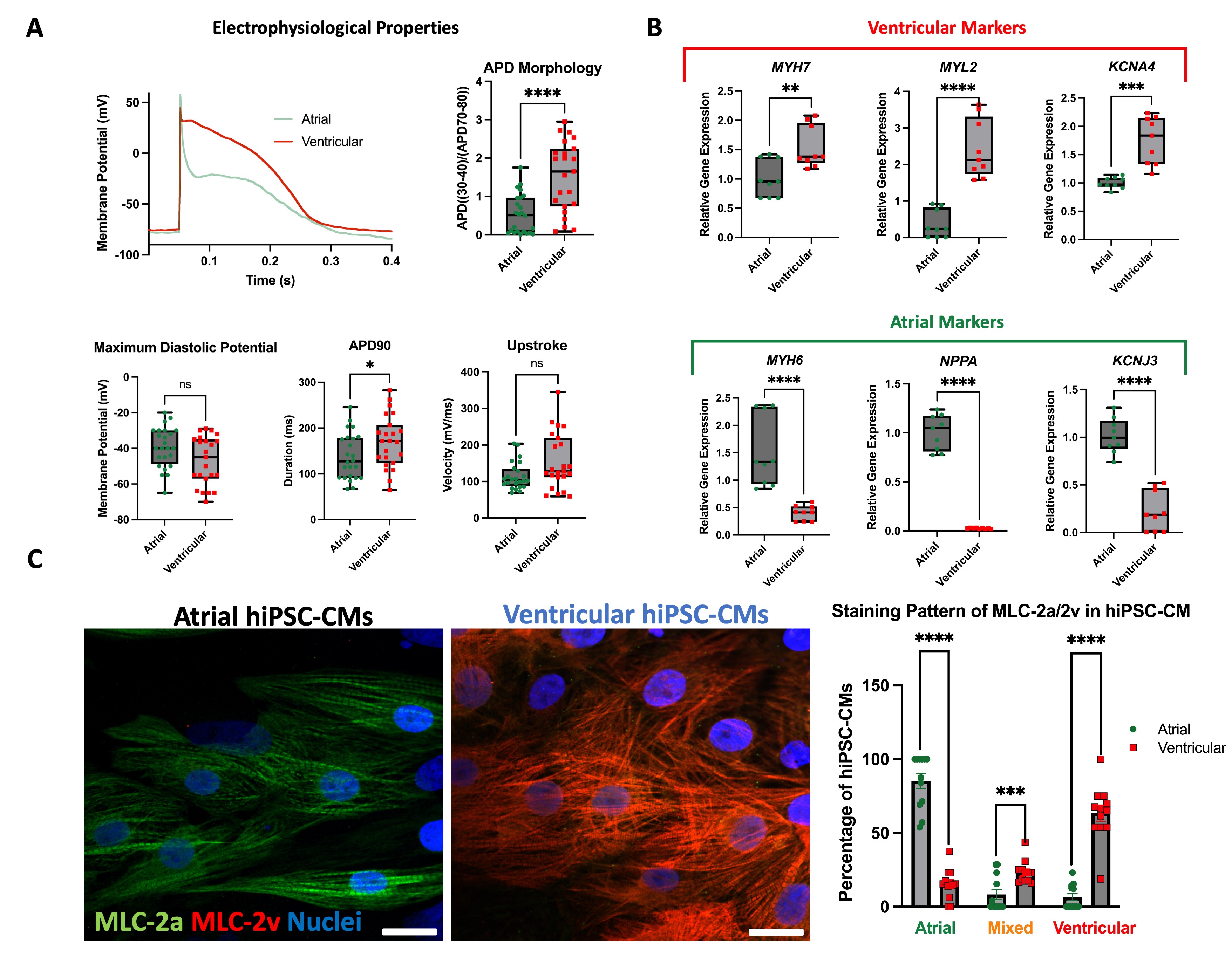

Supplement: cvag112_Supplementary_Data [file cvag112_supplementary_data.zip › Figure S5.jpg]

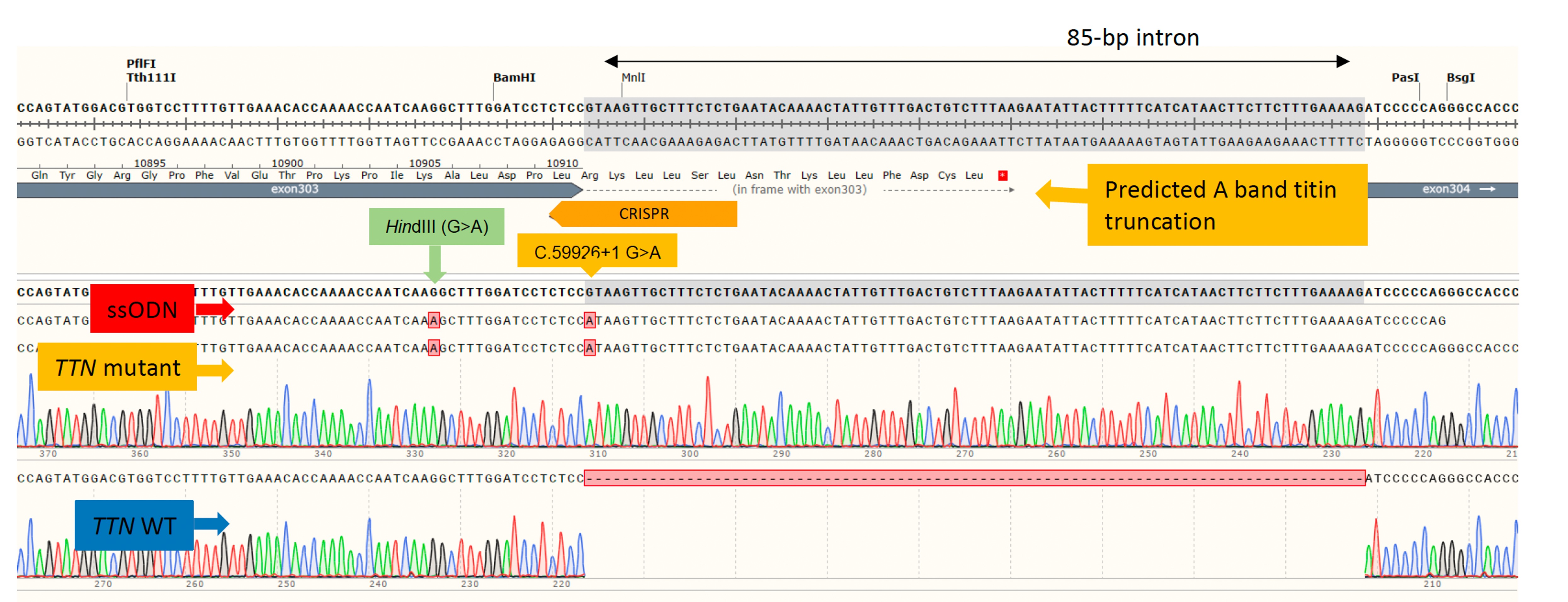

Supplement: cvag112_Supplementary_Data [file cvag112_supplementary_data.zip › Figure S6.jpg]

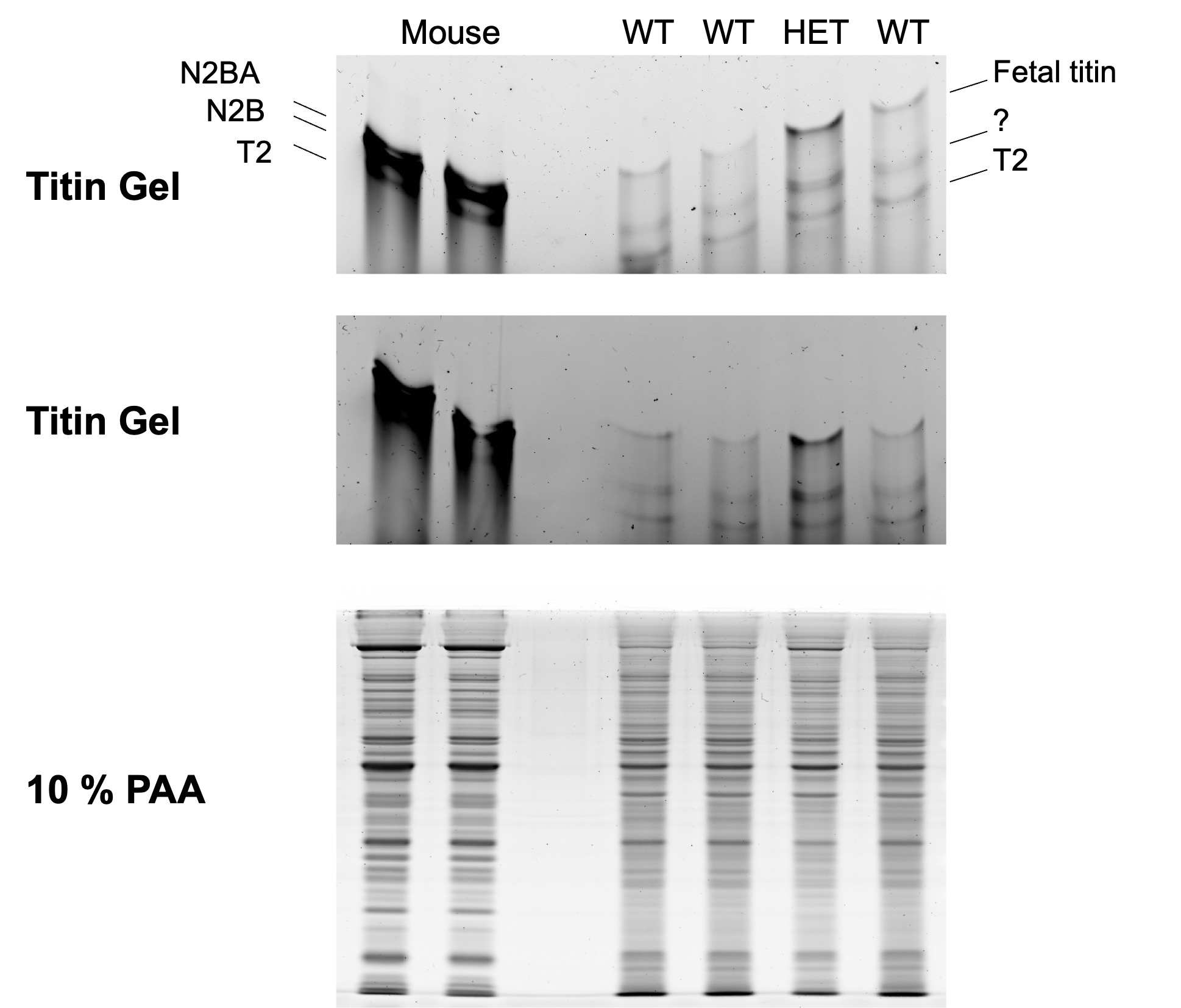

Supplement: cvag112_Supplementary_Data [file cvag112_supplementary_data.zip › Figure S7.jpg]

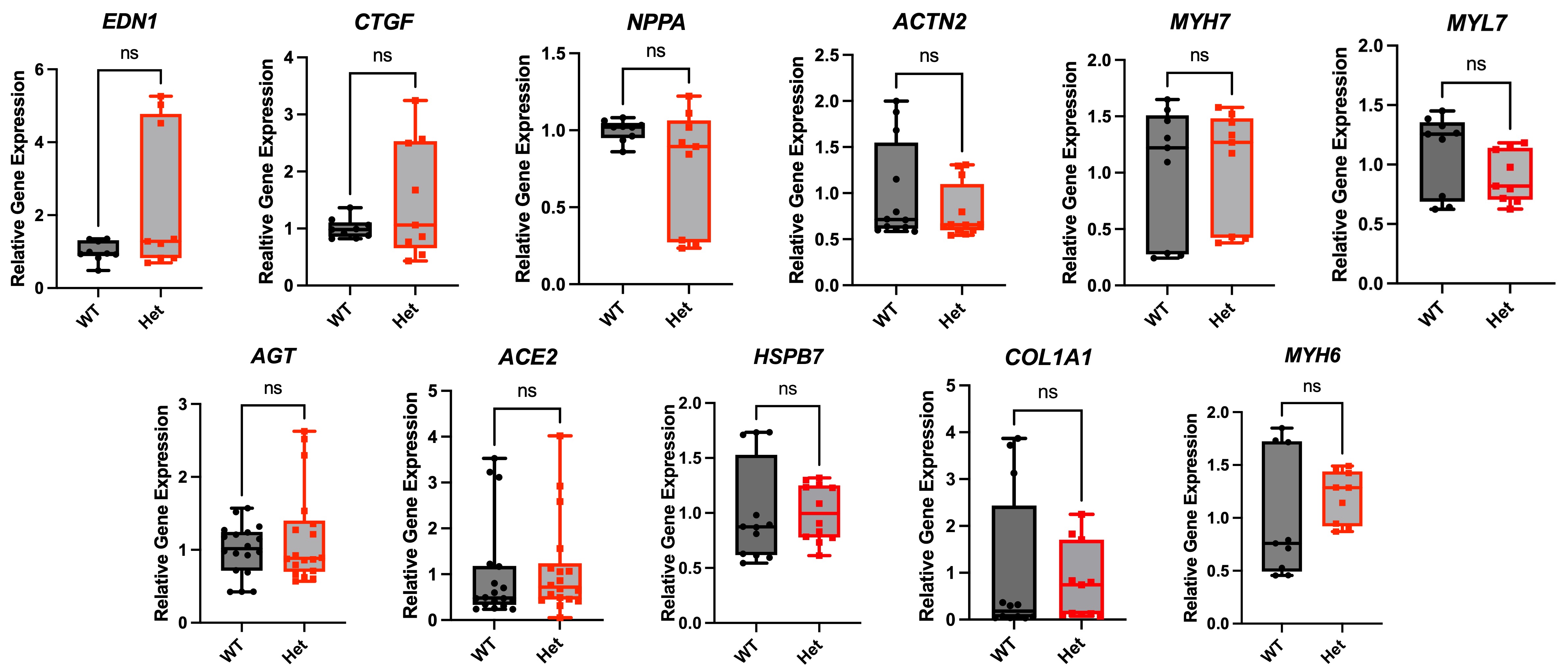

Supplement: cvag112_Supplementary_Data [file cvag112_supplementary_data.zip › Figure S8.jpg]

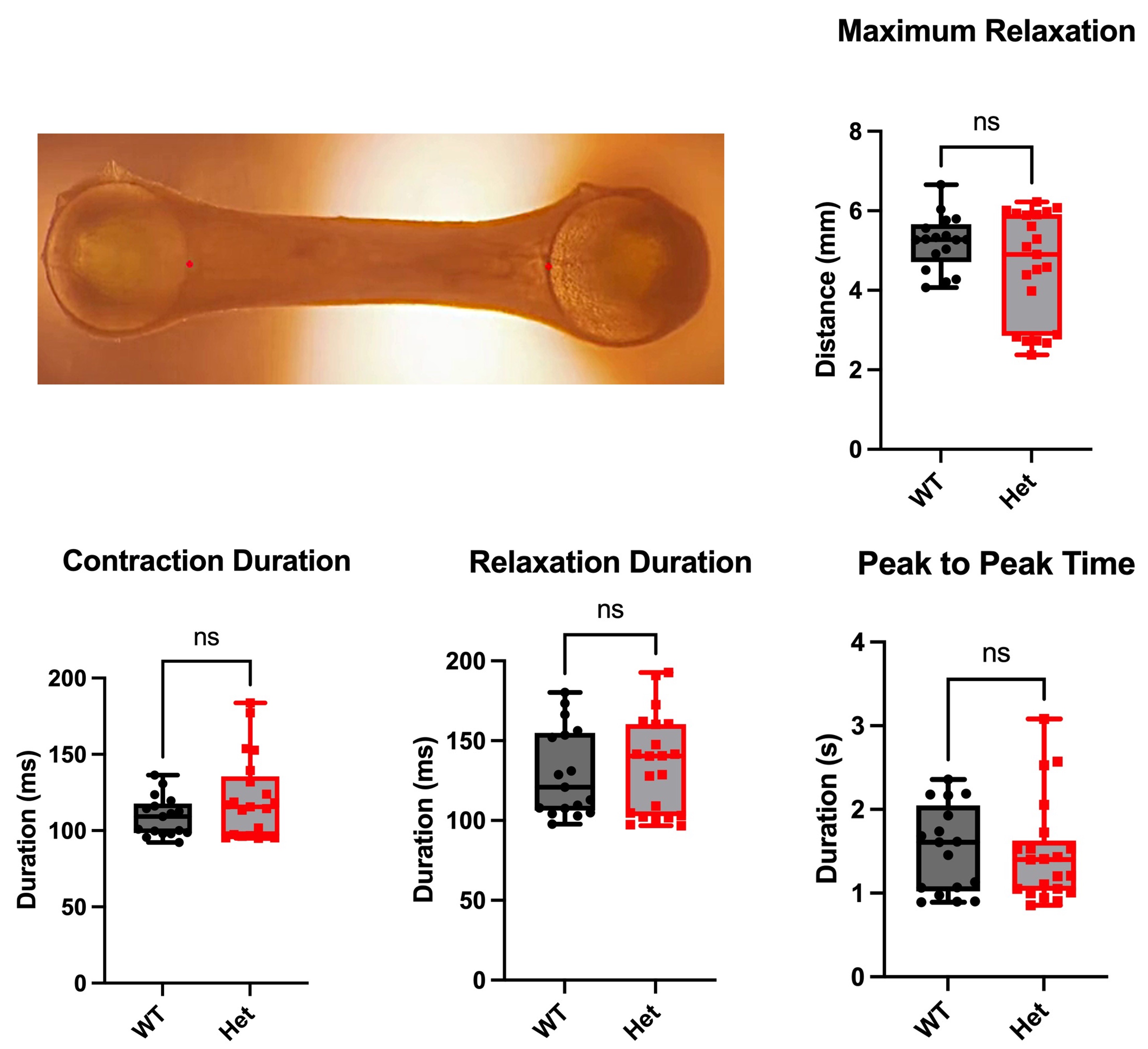

Supplement: cvag112_Supplementary_Data [file cvag112_supplementary_data.zip › Figure S9.jpg]

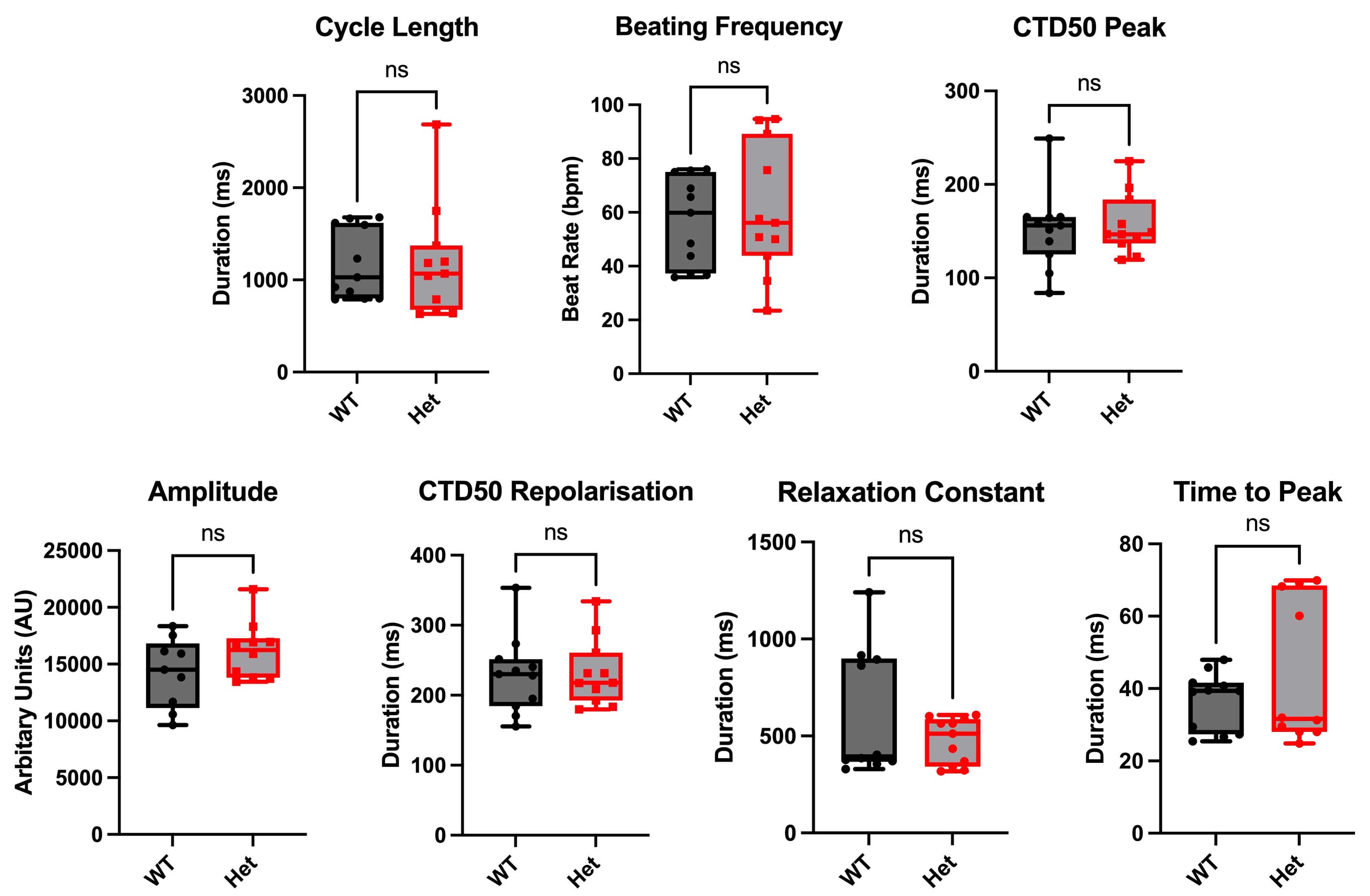

Supplement: cvag112_Supplementary_Data [file cvag112_supplementary_data.zip › Updated Figure S15.jpg]
